# Composition, Respirable Fraction and Dissolution Rate of 24 Stone Wool MMVF with their Binder

**DOI:** 10.1186/s12989-017-0210-8

**Published:** 2017-08-07

**Authors:** Wendel Wohlleben, Hubert Waindok, Björn Daumann, Kai Werle, Melanie Drum, Heiko Egenolf

**Affiliations:** 1 Department Material Physics and Analytics, BASF SE, Ludwigshafen, Germany; 2 Department of Aerosol Technology, BASF SE, Ludwigshafen, Germany

**Keywords:** Man-made vitreous fibres, Stone wool, Occupational safety, Biopersistence, Dissolution, Binder, Coating, Gel, Respirable fraction

## Abstract

**Background:**

Man-made vitreous fibres (MMVF) are produced on a large scale for thermal insulation purposes. After extensive studies of fibre effects in the 1980ies and 1990ies, the composition of MMVF was modified to reduce the fibrotic and cancerogenic potential via reduced biopersistence. However, occupational risks by handling, applying, disposing modern MMVF may be underestimated as the conventional regulatory classification -combining composition, in-vivo clearance and effects- seems to be based entirely on MMVF after removal of the binder.

**Results:**

Here we report the oxide composition of 23 modern MMVF from Germany, Finland, UK, Denmark, Russia, China (five different producers) and one pre-1995 MMVF. We find that most of the investigated modern MMVF can be classified as “High-alumina, low-silica wool”, but several were on or beyond the borderline to “pre-1995 Rock (Stone) wool”. We then used well-established flow-through dissolution testing at pH 4.5 and pH 7.4, with and without binder, at various flow rates, to screen the biosolubility of 14 MMVF over 32 days. At the flow rate and acidic pH of reports that found 47 ng/cm^2^/h dissolution rate for reference biopersistent MMVF21 (without binder), we find rates from 17 to 90 ng/cm^2^/h for modern MMVF as customary in trade (with binder). Removing the binder accelerates the dissolution significantly, but not to the level of reference biosoluble MMVF34. We finally simulated handling or disposing of MMVF and measured size fractions in the aerosol. The respirable fraction of modern MMVF is low, but not less than pre-1995 MMVF.

**Conclusions:**

The average composition of modern stone wool MMVF is different from historic biopersistent MMVF, but to a lesser extent than expected. The dissolution rates measured by abiotic methods indicate that the binder has a significant influence on dissolution *via* gel formation. Considering the content of respirable fibres, these findings imply that the risk assessment of modern stone wool may need to be revisited based on in-vivo studies of MMFV as marketed (with binder).

**Electronic supplementary material:**

The online version of this article (doi:10.1186/s12989-017-0210-8) contains supplementary material, which is available to authorized users.

## Background

Man-made vitreous fibres (MMVF) are non-crystalline, fibrous inorganic substances (silicates) made primarily from rock, slag, glass or other processed minerals. These materials, also called man-made mineral fibres, [1] include glass fibres (used in glass wool and continuous glass filament), rock or stone wool, slag wool and refractory ceramic fibres [2]. They are widely used for thermal and acoustical insulation and to a lesser extent for other purposes. These products are potentially hazardous to human health because they release airborne respirable fibres during their production, use and removal [3]. Fibre pathogenicity probably originates from a common mode of action from all respirable fibres, [4, 5] and is determined predominantly by aspect ratio, length and biopersistence [6, 7]. The traditional rock (or stone) wool was classified by the World Health Organization as a carcinogenic hazard to humans in 1988 [3]. In response, glass and stone wool compositions with increased biosolubility have been developed and commercialized [8]. Based on the in vivo tests required by the nota Q of the European CLP regulation, certain classes of MMVF are exonerated from classification as (carc. 2) carcinogen, [9] in accord with the conclusions of the World Health Organization report of 2002 [10]. Baan et al. very concisely review the considerations of the respective IARC Monographs Working Groups (1987, 2001) in reaching their conclusions [11]. In order to ensure that the increased biosolubility is maintained, the European insulation wool manufacturers association (EURIMA) implemented monitoring schemes to ensure that the chemical compositions are kept within defined ranges [12].

However, products should be tested as commercialized. The MMVF production inherently uses organic oil and binder (phenolic resin etc.) that is sprayed onto the stone melt directly in the fibre spinning chambers. The primary mat is layered to give the product the required weight per unit area, and passes through an oven, which sets the thickness of the mat, dries it and cures the binder [13]. The product is then air-cooled and cut to size before packaging [2]. Thus, MMVF without binder is not a necessary intermediate of occupational or commercial relevance. MMVF without binder is not representative of the commercial MMVF product for which safe use on construction sites must be ensured. Studies in 1995 deliberately removed binder from the commercial product, e.g. by ozone cold-ashing, [14] and only then investigated biopersistence. For the in vivo studies reported in 2000 – 2002, which were decisive for the WHO and IARC committees to exonerate the class of high-alumina low-silica stone wool (synonymously: HT, biosoluble MMVF) from classification as cancerogens, “tested fibres were produced without binder or oil” [15–17]. The conclusions and comments to the extensive BIA report^1^ already raise concerns that both in vitro data on dissolution and in vivo data on clearance and effects relate to MMVF without binder “that is rare in occupational settings” [18].

Here we took a pragmatic perspective motivated by occupational safety on BASF construction sites: We sourced MMVF directly from construction sites, and investigated their properties without further modifications. The strategy of the present contribution is to screen the safety-relevant physical-chemical properties –composition, respirable fraction, in vitro dissolution rates– on a set of modern stone wool MMVF sourced from various countries and producers. To the best of our knowledge, this is the first study to report composition, respirable fraction and dissolution of modern MMVF with their binder. We aim to benchmark results against literature on reference materials, which represent the low-biosolubility (MMVF21) and high-biosolubility (MMVF34) materials, respectively. Methodology for MMVF dissolution screening does not need to be re-invented, because it already is highly established [14, 19–21]. The strong correlation of in vitro dissolution rates vs. in vivo clearance rates, fibrogenic and carcinogenic potential was instrumental to identify safer MMVF compositions in the 1990ies [10, 22].

## Materials and Methods

**MMVF** were sampled in kg quantities predominantly from various construction sites within BASF Ludwigshafen, where contractor and/or BASF-employed workers handled MMVF products. Additionally, selected materials were sourced from sites in other countries, incuding Finland, UK, Denmark, Russia, China. In all cases, the producer and product grade are known, but are coded here for anonymity. Producers are coded A to E, and materials are coded MMVF #1 to #28 (due to multiple determination of oxide composition, some materials have more than one internal MMVF code, but are listed only once here.) Only one material (MMVF #17) cannot be traced to a specific grade and producer, because it was sampled from the dismantling of a BASF air separation facility, where it is known to have been installed at least for 30 years, hence with certainty before 1995. Thickness of all MMVF ranged from 40 to 150 mm, and density ranged from 47 to 180 kg/m^3^. Samples for dissolution testing were taken from the middle. Note that two most relevant historical reference materials are designated by the established codes MMVF21 and MMVF34. There is no specific relation between the historical reference MMVF21 and the modern MMVF#21.

**Sample pretreatment for Al, Ba, Ca, Cr, Mg, Mn, P, S, Sr, Ti composition analysis:** Analysis was performed for all materials in duplicate. In each case, a blank was run in an analogous manner. A sample aliquot of approx. 20 mg was weighed, to the nearest of 0.01 mg, into a platinum crucible, and mixed with both 0.8 g of a K_2_CO_3_-Na_2_CO_3_ mixture and 0.2 g Na_2_B_4_O_7_. The crucibles were inductively heated to a maximum temperature of approx. 930 °C. During the melt fusion, the crucibles were rotated and tilted to obtain a homogeneous melt. After cooling the melt cake was dissolved in approx. 88 ml of water and 12 ml semiconc. hydrochloric acid. The solution obtained was weighed again, and the final volume was calculated from the density of the solution (1.015 g/ml).

**Measurement of Al, Ba, Ca, Cr, Mg, Mn, P, S, Sr, Ti:** The analytes were determined by inductively coupled plasma-optical emission spectrometry (ICP-OES, Varian 725-ES). Prior to taking the measurement, the instrument was optimized in accordance with the manufacturer’s specification. Three replicate measurements are taken and averaged. We measured with 10 s integration time the following wavelengths [nm]: Al 396.152; Ba 493.408; Ca 317.933; Cr 206.158; Mg 279.553; P 213.618; S 181.972; Sr 216.596; Ti 336.122. The dilution factors were 6.67 for Al, Ba, Ca, Cr, Mg, Mn; and 1 for P, S, Sr, Ti. External calibration used concentrations of 0 / 1 / 5 mg/l with matrix-matched standards. The nebulizer (Meinhard 1 ml) had a flow of 0.7 l/min at pump rate 15 rpm. Complete reproduction on MMVF#4 through #11 confirmed better than 0.5% reproducibility on SiO_2_ and Al_2_O_3_ contents.

The slightly different sample preparation and measurement methods for optimal analysis of B (by ICP-OES) and Na, K (by Flame Atomic Absorption Spectrometry (F-AAS)) are described in full detail in the Additional file 1.

**Binder content and optional removal:** As a standard, all MMVF were measured as-received, without any sample preparation. For comparison in selected cases, the binder was removed by low thermal annealing or by oxygen plasma. Specifically, the oxygen plasma was generated in a Diener electronic PCCE, using 10 min at 60 W O_2_ plasma. Alternatively, an oven (Heraeus thermicon T), pre-heated to 500°C, was used to remove binder on samples of edge length 10 cm. The gravimetric loss can be accurately detemined on such large samples, and is attributed to the organic phase (binder). Full TGA on selected materials confirmed that 500°C is appropriate to remove binder. TGA utilized STA449 F3 (Netzsch), operated under air with 40ml/min, heated by 5K/min from 35 °C to 560 °C. The analysis software (Netzsch Proteus Thermal Analysis 6.1) adheres to DIN51005.

**Scanning Electron Microscopy (SEM)** was detemined both before and after dissolution testing. SEM samples were fixed on an adhesive film, coated with 9nm Pt and investigated on a JSM 7500TFE (Jeol Company) operated at 5 keV. The topographic images were taken with secondary electrons (SE).

**The BET specific surface area** was determined on Quantachrome Autosorb according to ISO 9277:2010 by volumetric static measurement of the nitrogen isotherm at 77.3 K with data evaluation according to the BET theory in the relative pressure range p/p_0_ between 0.001 and 0.3. Samples were prepared for adsorption analysis in a degasser, here the samples were heated up to 200 °C under vacuum for 30 min or more to remove moisture and other contaminations. For the specific equipment, we verified that specific surfaces down to 0.1 m^2^/g can be accurately determined. This was confirmed by repeatedly measuring Certified Reference Materials (Community Bureau of Reference - BCR 169 Alumina, certified at 0.100 m^2^/g, measured 0.095 m^2^/g; BCR 175 Tungsten, certified at 0.180 m^2^/g, measured 0.185 m^2^/g). However, the Certified Reference Materials have high powder density, whereas MMVF is less dense, resulting in a limited accuracy of the BET values of MMVF, which can deviate ± 0.15 m^2^/g, corresponding to about 30% uncertainty.

**Respirable fractions** To simulate handling of MMVF, between 100 g and 400 g were cut in an Alpine LU 100 rotating mill at around 1 kg/h throughput with an Ultraplex rotor at 94 m/s relative speed against a Conidur 0.2 mm sieve. Particle size distribution of the resulting MMVF dust was determined by cyclone cascade measurements. Dust samples were dispersed in a dosing feeder (K-Tron) and an injector with the aid of pressurized air (20 m^3^/h). 20 m^3^/h particle loading gas flow was fed into a 40mm tube with a length of 1m (Additional file 1: Figure SI 1). A part of the gas flow (1.7 m^3^/h) was sampled through a cyclone cascade. The separation cut-off size of the cyclone cascade is sub-divided in four steps from 10 μm to 0.3 μm. The aerodynamic diameter is defined as the diameter of a sphere with the density of 1 g/cm^3^ which has the same separation behavior as the measured sample. The adaption is conducted in accordance with the following equation:

$$ {d}_{ae}={d}_g\sqrt{\frac{\rho_g}{\rho }} $$

With d_ae_ = aerodynamic diameter, d_g_ = measured particle size, *ρ* = density 1 g/cm^3^, *ρ*_g_ = density of the sample substance. Weighing of the respective particle masses deposited on the individual cascade stages reflects the particle size distribution of the samples.

**Dissolution** testing replicated closely (Additional file 1: Figure SI 2) the well-established MMVF dissolution methods of Guldberg, Sebastian, de Meringo et al. [14, 19–21] as discussed extensively by BAuA [22] and by the BIA report [18]. The method is a dynamic (flow-through) system (Additional file 1 **:** Figure SI_2). Specifically, the amount of fibres (as a standard, m = 50 mg) is weighed with an accuracy of ±0.2 mg and is dispersed evenly in the cell. The measured BET *specific* surface area gives the *tested* surface area SA = m * BET. The flow rate was V = 48 ml/d, but was varied up to 240 ml/d. This corresponds to a ratio of the initial surface area SA to volume flow V of SA/V = 83 h/cm on average (min 38 h/cm, max 160 h/cm; in inverse metrics our average is V/SA = 0.033 μm/s). As we screened up to 7 cells in parallel, controlled by the same peristaltic pump (Ismatec IPC 8, Additional file 1 **:** Figure SI 2), the different BET specific surface areas of the materials result in slightly different SA/V. All testing performed at 37 ± 0.5 °C. The effluent was collected and pH checked. The programmable sampler drew 10 mL for ICPMS analysis (with the exact weight of each sample documented) after 1, 2, 4, 6, 8, 11, 14, 18, 21, 25, 28 and 32 days. This includes the sampling times of Guldberg et al. [14] and adds more to increase resolution and duration. Additionally to earlier methodology, we also collected eluted medium between the sampling times (Additional file 1: Figure SI 2). This enables a cumulative analysis including all dissolved ions, and becomes independent of interpolation. After the experiment the remaining fibres are rinsed in de-ionized water and dried to constant weight. The weight loss is compared to the value calculated from interpolation of the time resolved sampling and to the cumulative dissolved ions. The morphology of the corroded fibres in relation to the initial fibres is inspected by means of SEM.

**ICPMS and/or ICPOES** was used to determine dissolved ions in the eluates. All samples were analyzed for Si, Al and Mg. The between-sampling collection was analyzed for Si, Al, Mg, K, Ti, Fe, Ca. The samples were filtrated and diluted by a factor of 2 with de-ionized water. A higher dilution (factor 25) was used for elements with higher assays (Ca, K). A blank was run in an analogous manner. In the dilutions obtained, the analytes were determined by inductively coupled plasma-optical emission spectrometry (ICP-OES Agilent 5100). Prior to taking the measurement, the instrument was optimized in accordance with the manufacturer’s specification. Three replicate measurements are taken and averaged. We measured with 10 s integration time the following wavelengths [nm]: Al 394.401; Fe 259.940; K 766.491; Si 288.158; Ti 334.941 (axial observation); and Ca 396.847; Mg 279.553; (radial observation). The internal standard was Sc at 361.383 nm. External calibration used concentrations of 0 / 1 / 5 mg/l. The nebulizer (Meinhard 1 ml) had a flow of 0.7 l/min at pump rate 15 rpm. The analysis was performed in duplicate with less than 10% difference as reproducibility criterion. Otherwise, the analysis was repeated. The statistical error in all ion concentrations used for calculation of dissolution rates is thus below 10%.

**The pH 4.5 medium** composition, aiming to simulate the phagolysosome, replicated the “PSF” medium previously validated for the purpose of inhaled particle dissolution by NIST laboratories: [23] sodium phosphate dibasic anhydrous (Na_2_HPO_4_) 142.0mg/l; sodium chloride (NaCl) 6650 mg/l; sodium sulfate anhydrous (Na_2_SO_4_) 71 mg/l; calcium chloride dihydrate (CaCl_2_ . 2H_2_O) 29 mg/l; glycine (C_2_H_5_NO_2_) 450 mg/l (as representative of organic acids); potassium hydrogen phthalate (1-(HO_2_C)–2-(CO_2_K)–C_6_H_4_) 4085 mg/l; alkylbenzyldimethylammonium chloride (ABDC) 50ppm (added as an antifungal agent). This medium is near-identical to medium “C” in a previous interlab comparison of MMVF dissolution at pH 4.5 [19]. The pH 4.5 ± 0.4 was verified before and after the experiment, and was re-measured also on the eluted samples. Analysis of blind cells showed that Si and Al elements are sufficiently rare in the pH 4.5 medium, whereas the background levels of Ca interfere with the MMVF analysis.

**The pH 7.4 medium** composition, aiming to simulate the extracellular lung compartment, followed one of the previously described Gamble’s fluids. [24] magnesium chloride (MgCl_2_) 95 mg/l; sodium chloride (NaCl) 6,019 mg/l; sodium phosphate dibasic anhydrous (Na_2_HPO_4_) 298 mg/l; sodium sulfate anhydrous (Na_2_SO_4_) 63 mg/l; calcium chloride dihydrate (CaCl_2_ . 2H_2_O) 368 mg/l; sodium acetate (C_2_H_3_NaO_2_) 574 mg/l; sodium hydrogen carbonate (NaHCO_3_) 2,604 mg/l; sodium citrate dihydrate (Na_3_C_6_H_5_O_7_) 97 mg/l. We added sodium azide (NaN_3_) 20 mg/l as biocide. The MMVF literature documents a variety of Gamble’s pH 7.4 fluids, and the present version is consistent with others used earlier on MMVF dissolution [20]. The pH 7.4 ± 0.3 was verified before and after the experiment, and was re-measured also on the eluted samples.

## Results

### Composition

The oxide composition is summarized in Table 1, listing the 15 MMVF materials that were also subjected to dissolution screening. All products tested were within a narrow range of SiO_2_ content, ranging from 40% to 44%, with an average of 42% SiO_2_ of the inorganic part. Additionally to the inorganics, organic components were detected in all MMVF with a content of the total weight from 0.9% to 4.2%. In TGA, the mass loss occurs in peaks between 300°C and 500°C, with an average mass loss of 2.8 ± 1.0 % below 500°C. The organic component is identified with the binder, oil, resins etc. that coat the surface of the fibres. The binder is observed also on SEM micrographs, where it visibly glues fibres together. Detailed analysis of binder chemical composition was not performed. By SEM, the distribution of fibre diameter is polydisperse with diameters from below 2 μm to above 20 μm, and often included large nonfibrous “shot” particles on the order of 200 μm. Thus, the majority of fibres in MMVF is too large to penerate deep into the lung, but every modern MMVF examined did have a small fraction of respirable fibres. Full statistical analysis of the fibre diameter distribution was beyond the scope of the present contribution, but the following section addresses the airborne fibre fraction. Finally, the specific surface area SA of the MMVF ranged between 0.2 and 0.6 m^2^/g. For orientation, using the density of 2.8 g/cm^3^ and the known fibre shape, the SA can be converted to an average diameter of the fibres, giving values between 2.4 μm and 10 μm. Considering the polydispersity, this is consistent with SEM and with literature [10].

**Table 1:** Composition of MMVF sourced from various countries and producers. Weight content of oxides and of binder

Country of origin	Producer code	MMVF code	SiO_2_	Al_2_O_3_	CaO	MgO	Fe_2_O_3_	TiO_2_	Na_2_O	K_2_O	MnO	P_2_O_5_	Cr_2_O_3_	BaO	S	SrO	B_2_O_3_	SUM	Al / (Al+Si)	KI-Index	BET (m^2^/g)	% binder content
Germany	unkown	MMVF #17 (pre-1995)	54	7	30	3	3	0.5	1.5	1.3	0.1	0.2	0.1	0.0	0.1	0.1	0.0	100	0.13	22	0.3	0.1
Germany	A	MMVF #1	42	18	18	9	7	2.1	2.1	1.2	0.4	0.4	0.1	0.1	0.1	0.1	0.0	101	0.33	-6	0.2	4.2
Germany	B	MMVF #2	42	18	18	9	8	1.8	2.4	0.9	0.2	0.3	0.1	0.1	0.1	0.1	0.0	102	0.38	-5	0.5	4.1
Germany	C	MMVF #4	44	24	15	2	6	0.7	6.2	3.8	0.2	0.8	0.1	0.1	0.0	0.1	0.0	102	0.32	-21	0.6	1.1
Germany	A	MMVF #5	43	18	17	9	7	1.9	1.3	0.7	0.3	0.3	0.1	0.1	0.1	0.1	0.0	99	0.34	-8	0.2	3.9
Germany	A	MMVF #7	40	20	16	10	1	1.2	2.3	2.2	0.9	0.3	0.3	1.0	0.1	0.1	0.1	95	0.36	-7	0.4	2.4
Germany	A	MMVF #8	41	19	18	10	8	1.4	0.8	0.4	0.7	0.4	0.3	0.1	0.1	0.1	0.0	100	0.34	-8	0.2	0.9
China	A	MMVF #11	42	19	19	8	7	1.3	1.4	0.3	0.2	0.1	0.0	0.1	0.1	0.1	0.0	98	0.35	-8	0.3	4.1
Germany	A	MMVF #12	43	20	19	10	7	1.9	2.6	1.4	0.4	0.3	0.1	0.1	0.1	0.1	0.0	105	0.34	-7	0.2	3.7
Germany	D	MMVF #14	44	16	23	9	6	1.6	2.7	1.7	0.6	0.3	0.1	0.2	0.1	0.1		105	0.35	6	0.5	3.7
Germany	E	MMVF #20	42	18	18	13	8	0.8	1.4	0.4	0.1	0.1	0.1	0.1	0.1	0.1	0.0	101	0.36	-2	0.2	1.9
Finland	E	MMVF #21	42	17	16	12	10	0.8	1.5	0.4	0.1	0.1	0.1	0.1	0.1	0.1	0.0	100	0.29	-4	0.4	2.4
UK	A	MMVF #22	40	17	22	9	9	1.3	2	0.4	0.4	0.2	0.1	0.1	0.3	0.1	0.0	102	0.30	-1	0.4	3.2
Russia	A	MMVF #24	41	16	24	9	8	1.3	1.3	0.7	0.3	0.1	0.1	0.1	0.5	0.1	0.0	102	0.34	2	0.4	2.4
Germany	A	MMVF #26	41	19	19	8	7	2.1	2.7	0.7	0.2	0.4	0.1	0.1	0.1	0.1	0.0	100	0.34	-7	0.3	1.9

Nine additional MMVF materials from further countries and producers were analyzed for their composition, binder content, specific surface area and SEM morphology (available, not shown here). Their properties (summarized in Additional file 1: Table SI_1) are consistent and remain within the ranges observed on the main test set.

### Respirable fraction of milled MMVF

MMVF #2, #5, #12 were chosen for screening fractionation because their composition is roughly average across the entire test set, and thus considered to be representative. After milling, their dusts were dispersed and separated by the aerodynamic diameter by means of a cyclone. The weight collected on the impactor stages shows that for all MMVF investigated, about 59-75% of total dust mass has aerodynamic diameters >10μm, and are thus not or only partially inhalable to humans. However, the cyclone fractions with aerodynamic diameters below 7.4 μm vary from 0.29% to 6.29% of milled MMVF mass for the modern MMVF (Table 2). The spread of this fraction is large, but not different from the value of 3.65% found in the dust from historic MMVF#17 (Table 2). The next smaller fraction with aerodynamic diameters up to 4.2 μm is significantly lower with values from 0.02% to 0.22% for the modern MMVF, to be compared to 0.04% for the historic MMVF#17. Two of our modern MMVF were similar, but one (MMVF#5) has significantly lower dustiness, as it also was visually soft and clumpsy already during milling.

**Table 2:** Fractionation of airborne MMVF. Weight gain of the total initial MMVF mass per fraction

Impactor stages, aerodynamic diameters	MMVF #2	MMVF #5	MMVF #12	MMVF #17 (pre-1995)
< 7.6 μm	2.40%	0.29%	6.29%	3.65%
< 4.1 μm	0.22%	0.02%	0.13%	0.04%
< 1.2 μm	0.08%	0.01%	0.08%	0.01%
< 0.3 μm	0.03%	<<0.01%	0.04%	<<0.01%
< 0.1 μm	<<0.01%	<<0.01%	<<0.01%	<<0.01%

**Fig. 1:**
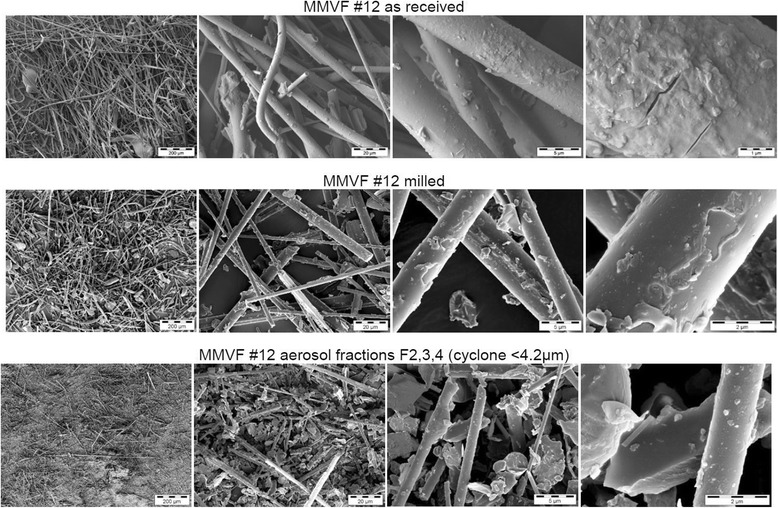
SEM micrographs of MMVF #12. As received – after milling – only respirable fraction. See the Supporting Information for analogous results on MMVF #5

SEM analysis confirms that the cyclone fractions consist of thinner fibres, and their diameters are consistent with the nominal aerodynamic diameter cut-offs (Fig. 1 and Additional file 1: Figure SI_3). The fractions also contain milling debris of short fragments with low aspect ratio.

### Dissolution

**Fig. 2:**
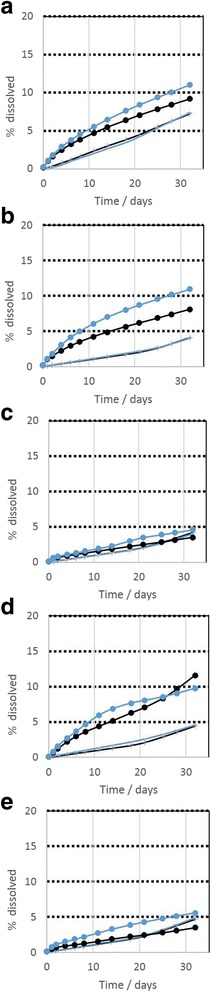
Dissolution kinetics in neutral and acidic pH conditions, all at initial mass 50 mg MMVF, flow 48 ml/day. Si (*black lines*), Al (*blue lines*). pH 4.5 (dots), pH 7.4 (crosses). **a** MMVF #4, **b** MMVF #5, **c** MMVF #12, **d** MMVF #14, **e** MMVF #22

Screening was performed on the as received MMVF materials. The ions detected at each sampling time are normalized to the initial content of the specific element in the specific MMVF, and are then interpolated with due consideration of the different sampling intervals to finally obtain the percentage that has dissolved from this oxide. The resulting kinetics are plotted in Fig. 2 for both pH conditions. In pH 7.4, dissolution is consistently accelerating over the 32 days of sampling, and Si and Al dissolve with near-identical kinetics, despite their very different content in these materials. In contrast, in pH 4.5 we observe a significantly faster dissolution. Further, in pH 4.5 the initial dissolution rate tends to slow down over time. Finally, at pH 4.5 we observe a higher fraction of Al than Si in ionic form (Fig. 2), and an even higher fraction of Mg (Additional file 1: Figure SI_4).

Additionally to the kinetics sampling, the entire elution medium is collected between the sampling times and analyzed so that *all* eluted ions are known. The sum of all ions provides the cumulated dissolved fraction, independently for Si and Al, which is then weighted by their relative content to give the “ion” columns in Table 3, divided by the initial surface area SA and by the total time of 32 days to obtain the dissolution rate k in units of ng/cm^2^/h. Table 3 summarizes the results for a total of 15 materials. For each dissolution experiment, the quantitative assessment by “ions” is supported by a complementary assessment by gravimetry of remaining solids, which is shown for the two main screenings in pH 4.5 and pH 7.4 at standard conditions as column “S” in Table 3.

**Table 3:** Dissolution screening at pH 7.4 and pH 4.5. “Ions” = cumulated dissolved Si and Al based on ICPMS quantification of all eluted ions, in % of initial Si and Al; “S” = remaining solid mass, in % of initial MMVF mass; “k” = dissolution rate determined from cumulated dissolved ions, in ng/cm^2^/h

	screening at pH 4.5			screening at pH 7.4			pH4.5, (binder removed)	pH4.5 (milled)	pH4.5 respirable (milled + cyclone)	pH4.5 (1/5 lower SA/V)	
	Ions (%)	S (%)	k	Ions (%)	S (%)	k	k	k	k	k	SEM morphology before and after after dissolution (full data in SEM Annex)
MMVF #17 (pre-1995)	2	95	9								Untreated – smooth surface
											pH4.5 - very limited change of fibre surface after treatment: minimal roughening, no significant gel formation, no leaching pits.
MMVF #1	11	85	90	4	94	31				171	Untreated - binder covers entire fibre, including 100 nm to 1 μm sized particles.
											pH4.5 - Very limited change of fibre surface after treatment: occasionally approx. 50 nm small leaching pits (pores).
											pH7.4 – significant change of fibre surface, approx. 200nm to 1 μm gel/deposit/crater structures
MMVF #2	9	96	23								Untreated - smooth surface, occasional lumps of 100 nm to 1 μm sized particles.
MMVF #4	10	100	20	7	92	17	39				Untreated – smooth surface
											pH4.5 – very significant change of fibre surface with pronounced gel; frequent occurence of approx. 400nm sized deep craters with sub-100-nm cracks at bottom.
											pH4.5 – without binder, change of fibre surface with limited gel, no cracks, no pits, but approx. 200nm sized shallow structures. Thermal removal more effectiove than plasma removal of binder.
											pH7.4 - very significant change of fibre surface with pronounced, inhomogenously structured gel and/or deposits, up to approx. 400nm large crater
MMVF #5	10	85	87	4	97	35		104	122		Untreated – smooth surface, occasional lumps of 100 nm to 1 μm sized particles.
											pH4.5 - significant change of fibre surface with extensive gel formation, leaching with sub-100-nm sized pits and cracks. pH4.5 on respirable-only fraction also induces gel formation, leaching with sub-100-nm sized pits
											pH7.4 - significant change of fibre surface, inhomogeneous leaching through gel with 1-μm-diameter honeycomb structures.
MMVF #7	11	94	40	2	99	6	58				Untreated – smooth surface, occasional 200 nm sized particles
											pH4.5 – significant change of fibre surface with extensive gel formation, up to 4 μm sized leaching pits, occasional micro-cracks and approx. 100 nm large pores.
											pH4.5 – binder removed thermally: no significant gel formation, occasional micro-cracks (intermediate gel formation if binder removed by plasma)
											pH7.4 – very significant change of fibre surface with gel formation and/or deposits of up to approx. 2 μm large crystalline particles
MMVF #8	10	97	65								Untreated – smooth surface. Binder covers entire fibre (see ruptured crossings).
											pH4.5 –significant change of fibre surface with gel formation, numerous up to approx. 100 nm sized leaching pits (pores).
MMVF #11	13	85	58								Untreated – smooth surface, occasional lumps of 200 nm to 1 μm sized particles
											pH4.5 – very significant change of fibre surface with gel formation and deep micro-cracks (3μm x 300nm). Pitting (approx. 1 μm large spots) and approximately 200nm visible pores
MMVF #12	6	93	36	4	94	27		31	29		Untreated – rough surface, lateral cracks
											pH4.5 – significant change of the fibre surface, increased smoothness. No significant gel formation, no leaching pits; pH 4.5 on respirable-only fraction: limited gel formation, no significant leaching pits.
											pH7.4 – significant change the fibre surface with pronounced gel formation, inhomogenous leaching, approx. 4μm x 0.5μm sized leaching pits
MMVF #14	8	90	24	4	93	12					Untreated – binder covers entire surface, frequent inclusion of approx. 100 nm sized particles
											pH4.5- significant change of fibre surface with gel formation, inhomogenous leaching, up to approx. 500 nm large leaching pits, occasionally up to approx 200 nm wide pits/pores.
											pH7.4 – very significant change of fibre surface with fine grained deposits of approx. 100 to 500 nm sizes.
MMVF #20	7	89	49								Untreated – smooth surface, binder covers entire surface
											pH4.5 – significant change of fibre surface with gel formation and erosion by frequent approx. 50 nm sized leaching pits/pores
MMVF #21	7	99	23								Untreated – smooth surface, very rare inclusion of approx. 200 nm sized particles
MMVF #22	5	91	17	5	95	15					Untreated – smooth surface with inclusion of approx 250 nm sized particles.
											pH4.5 – significant change of fibre surface with gel formation; up to 1.5 μm diameter plateaus that have an up to 1 μm long micro crack in their center.
											pH7.4 – significant change of fibre surface with gel formation, approx. 500nm diameter leaching crater
MMVF #24	9	91	28								Untreated – smooth surface with occasional inclusion of approx 500 nm particles
											pH4.5- very significant change of fibre surface with 2 μm diameter plateaus with several approx 50 nm leaching pits on the plateaus
MMVF #26	4	93	18								Untreated – structured surface with approx. 200 nm to 1μm diameter elevations.
											pH4.5 – significant change of fibre surface with gel formation, no leaching pits.

**Fig. 3:**
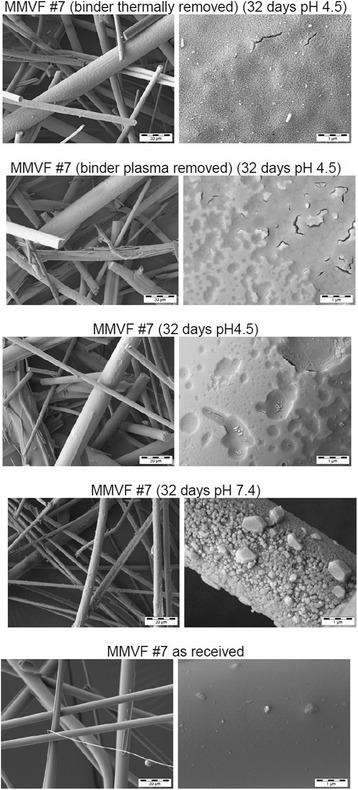
MMVF #7 (average composition MMVF) morphology by various dissolution conditions at pH 4.5 and pH 7.4, with and without binder: SEM analysis

**Fig. 4:**
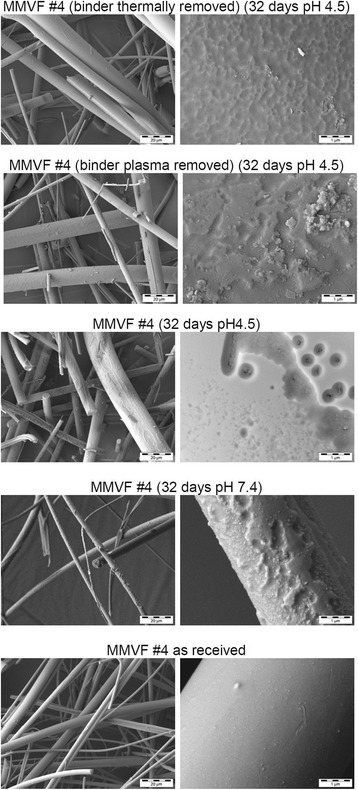
MMVF #4 (high-alumina) morphology by various dissolution conditions at pH 4.5 and pH 7.4, with and without binder: SEM analysis

The remaining solids were further imaged by SEM, and compared against the MMVF before aging (which is not the identical sample taken for dissolution, as SEM typically requires coatings). We make no attempt to evaluate statistically the fibre diameters. Instead, the SEM analysis shows that in general the untreated MMVF surface is smooth with occasional inclusion of 100 nm to 1 μm sized particles. Judging from the morphology at fibre junctions, the binder covers the entire MMVF surface (see untreated fibres in Additional file 1: Figure SI_5, especially MMVF #8, #14, #20). The re-analysis by SEM after dissolution confirms that the fibre morphology is persistent after aging, in general without splicing or obvious shortening. For exemplary detailed results, MMVF #7 (Fig. 3) was chosen because its composition is roughly average across the entire test set, and thus considered to be representative. MMVF #4 (Fig. 4) was chosen because it has the highest Al content of the entire test set and is an innovative product with process and benefit characteristics inherited from both stone wool and glass wool. MMVF #7 and MMVF #4 both change their surface significantly after 32 days at pH 4.5, showing pronounced gel formation. For instance on MMVF #4, deep craters of approx. 400 nm diameter with sub-100-nm cracks at their bottom are frequently observed after dissolution in pH 4.5 (Fig. 4). In contrast, after dissolution in pH 7.4, deposits that often appear crystalline are found on the surfaces. For further comparison Additional file 1: Figure SI_5 shows the near-absence of morphological changes on MMVF #17 (pre-1995), in excellent accord with the very low dissolution. Throughout the test set of modern MMVF, at pH 4.5 a gel with leaching structures in the form of pits is frequently observed, occasionally also leaching craters, plateaus, microcracks and also ring-shaped hems (potentially collapsed bubbles). Fibre breakage is rare. The SEM observations are summarized in Table 3, with high magnification scans shown in Additional file 1: Figure SI_5. Full SEM results are documented in the Additional file 2: SEM Annex with low-mid-high magnification for all MMVF before and after dissolution.

**Fig. 5:**
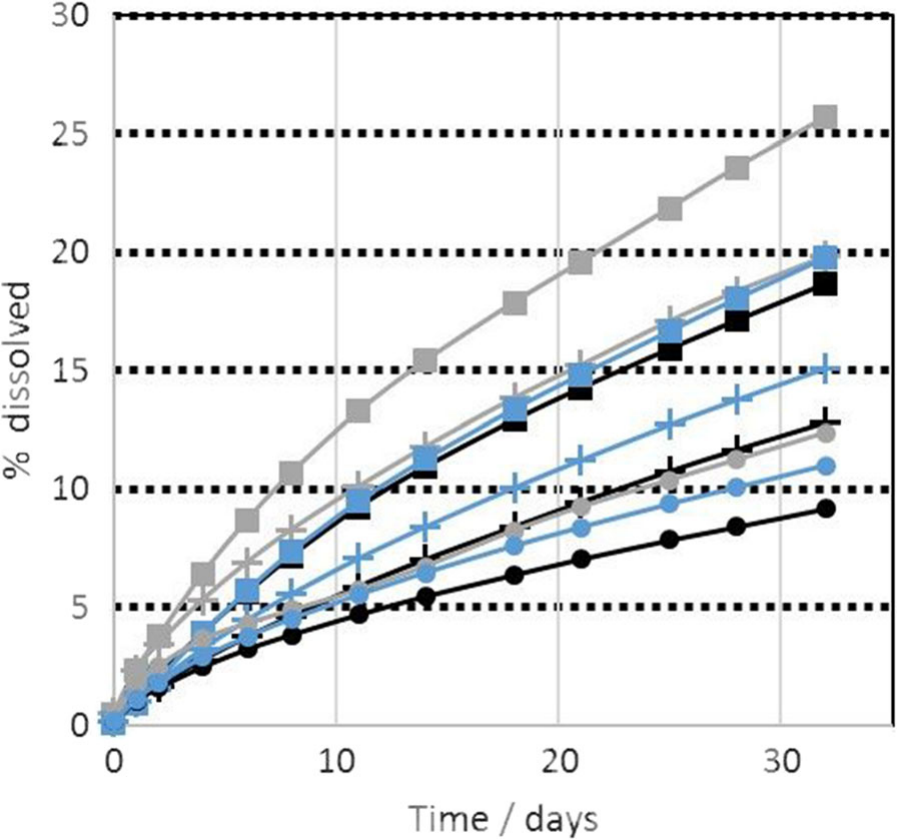
Binder effects on dissolution kinetics at pH 4.5 of MMVF #4, with binder (*dots*), binder removed by plasma (*crosses*), binder removed thermally (*boxes*) for the three oxides Si (*black*), Al (*blue*), Mg (*grey*)

Several materials were subjected to modifications of the standard conditions, in order to explore the origin of the unexpectedly low dissolution rates. To compare against literature, binder was removed by two different methods from MMVF #7 and from MMVF #4, which represent high and low binder content respectively. Then dissolution at pH 4.5 was performed and found a dramatic acceleration of Mg leaching and of Si, Al dissolution from the high-alumina fibre MMVF #4 (Fig. 5). The k rate based only on Si and Al doubles from 20 to 39 ng/cm^2^/h (Table 3), and the remaining solids even drop to 64% after 32 days. The effects are less pronounced but equally a significant acceleration from 40 to 59 ng/cm^2^/h is observed for MMVF #7. In accord, also the dissolution morphology changes without binder. The surface is much smoother with leaching pits reduced in size or completely absent. Plasma treatment has an intermediate effect both on morphology (Figs. 3 and 4) and on kinetics (Fig. 5a, b). We also performed nitrogen adsorption before and after binder removal on MMVF#4, #5 and #7 (low, high and mid binder content). The dimensionless BET isotherm fitting constant c reduces by a factor 2.8±1.3 with the binder. Due to this change of physisorption mechanisms, the net change of specific surface area by the presence of binder has positive or negative sign, depending on the evaluation model: -22% by BET evaluation, +15% by Langmuir evaluation.

Additional dissolution studies were also performed on the respirable fractions of MMVF #5 and MMVF #12, finding an increase of dissolution rates for MMVF #5 up to 122 ng/cm^2^/h, and an slight decrease for MMVF #12. For both cases, the milled, not fractionated materials had intermediate dissolution rates (Table 3).

## Discussion

### Composition

Man-made vitreous fibres (MMVF) are classified within the European Union (EU) as carcinogen category 2 (suspected human carcinogens), but Nota Q and Nota R specify criteria to exonerate fibres from this classification [9]. The HT stone wool fibres are a range of MMVF compositions that fulfill European regulatory requirements for exoneration from classification as a carcinogen and are registered by a chemical compositional range for the CAS number 287922-11-6. This range –synonymously designated as “High-alumina, low-silica wool” or “HT stone wool” or “biosoluble stone wool”– is defined by the range of dominant metal oxides shown in Table 4, with silica in the range 33% – 43%, alumina in the range 18% – 24%. In contrast, the MMVF class of pre-1995 “Rock (stone) wool” has a significantly higher SiO_2_ content of 43% – 50%, and lower Al_2_O_3_ content of 6% – 15% [10]. As rationale for the delimitation of the HT class with high biosolubility, it has been proposed that “an increase in Al/(Al + Si) ratio will result in a more hydrated and less continuous remaining silica network as aluminum is removed [….] As a result, the ability to form dense surface layers is reduced and hereby the dissolution rate increases” [16]. The modern MMVF were analyzed to have a content of SiO_2_ highly controlled within a narrow range, and with still relatively similar contents of Al_2_O_3_, CaO_2_, MgO, showing in this order an increasing spread of composition across the test set. As defined at the time of introducing the HT fibres “a maximum limit of 43% SiO_2_ and a minimum limit of 18% Al_2_O_3_ and 23% CaO + MgO should secure that the fibres are biosoluble” [16]. The condition on Al_2_O_3_ is fulfilled by nearly the entire test set, but the conditions on SiO_2_ and CaO+MgO are frequently not fulfilled. Thus, most but not all of the present set of modern MMVF belonged to the class of “High-alumina, low-silica wool”. Part of the test set was on or beyond the borderline to the class of pre-1995 “Rock (Stone) wool”. In terms of the the Al/(Al+Si) ratio, and also in terms of SiO_2_ content, the average of the test set is halfway between the references MMVF21 and MMVF34, with a relatively wide spread of individual oxides, but a very low spread in the Al/(Al+Si) ratio = 0.34 ± 0.05 (min 0.29, max 0.38).

**Table 4:** SUMMARY of modern MMVF (#1 to #28) compared to IARC reference ranges: results on composition and dissolution. The compositional range of Rock (stone) wool (cancerogen classification, low biosolubility) is represented by the historical reference MMVF21. The compositional range of high-alumina, low-silica wool (synonymously HT stone wool *or* high biosolubility stone wool or CAS 287922-11-6) is represented by the historical reference MMVF34

	High-alumina, low-silica wool (exonerated from classif.)	MMVF34 (represents high biosolubility)	Rock (stone) wool, (cancerogen classification)	MMVF21 (represents low biosolubility)	This test set (excluding MMVF #17)		
COMPOSITION	[10]	[19]	[10]	[19]	Average	Min	Max
SiO_2_	33 – 43	39	43 – 50	46	42	40	44
Al_2_O_3_	18 – 24	23	6 – 15	13	19	15	24
CaO	23 – 33	15	10 – 25	17	28	16	33
MgO		10	6 – 16	9			
Fe-oxides	3 – 9	7	3 – 8	6	7	1	10
Al/(Al+Si)		0.41		0.25	0.34	0.29	0.38
DISSOLUTION					Average	Min	Max
k_Si_ pH 4.5 in ng/cm^2^/h	> 400 ^‡^	831 (620)*		47 (72)*	41	17	171
k_Si_ pH 7.4 in ng/cm^2^/h		58 (59)*		23 (20)*	20	6	35

*Note: Comparison of IARC Table 65 to Guldberg et al (1998) Table 4 clarifies that IARC chose to document for MMVF21 and MMVF34 the k_leach_ value, which reports Ca and Mg ions. The actual fibre disintegration is assessed by earlier and by the present studies via k_Si_. Hence we compare materials based on k_Si_ and provide the values from the IARC table in brackets for transparency [10, 19]

‡The value of k > 400 ng/cm^2^/h for the exonerated CAS range is from [16]

### Dissolution

The class of HT stone wool is specifically designed to have relatively lower solubility at neutral pH (for technical performance) and high solubility at acidic pH (for product safety). Lower solubility at neutral pH is advantageous for technical durability for the intended use. The alumina content, to replace silica, is advantageous to reduce costs and to increase productivity via having a melt viscosity at 1,400° C of 10—70 poise. Hence, even patents specify dissolution at pH 4.5, and define a particularly preferred class by SiO_2_ < 42.0% and Al_2_0_3_ > 20% [25]. Literature “supports the use of in vitro fibre degradation at pH 7.4 and/or pH 4.5 as an indicator of SVF [synthetic vitreous fibre] potential pathogenicity” [26]

We observe at pH 4.5 and also at pH 7.4 dissolution rates that are very similar to stone wool MMVF21, which is plausible considering the related composition. And yet, the decreased SiO_2_ content, halfway to the MMVF34 reference, should result in intermediate dissolution rates as well. MMVF21 at pH4.5, tested at same SA/V as in our screening, had k_Si_ = 47 ng/cm^2^/h, k_leach_ = 72 ng/cm^2^/h, and at pH7.4 a k_total_ = 23 ng/cm^2^/h [19]. The k_leach_ rate reports Ca and Mg ions whereas k_total_ integrates all measured ions. Focusing on the disintegration of vitreous fiber structure , this is an acceleration by 2.04 in acidic vs. neutral pH. For comparison, the average acceleration in our test set is 2.5. Considering the wide span from <<1 to >>1 for different MMVF types, this is a close match. Further, also for MMVF21 the moderate contribution of leaching with a 1.5 fold higher leaching rate than Si-based dissolution rate is fully consistent with our observations of 1.1 to 1.6 higher leaching rates (based on Mg) as compared to the Si-based dissolution rates.

In the following we systematically discuss potential sources of error in the dissolution methodology:

1. Earlier studies used sieved material without shot. We might have underestimated k by slow dissolution of thick shot particles. This hypothesis was tested experimentally here. Indeed, comparison of our dissolution of entire MMVF against our dissolution of respirable fraction shows in one case an acceleration but in another case moderate deceleration (Figure 1, Additional file 1: Figure SI_3, Table 3: +40% for MMVF #5, -20% for MMVF #12). Overall the shot effect and diameter effect are not enough to explain the slow dissolution. This is supported by Potter, finding only a 17% acceleration between MMVF34 and respirable-separate MMVF34 [27].
2. The media are not completely standardized throughout literature. Our pH 4.5 medium, also designated as “Phagolysosomal Simulant Fluid” (PSF) [23] is actually based on earlier MMVF media, and has the identical ingredients as the medium “C”, also designated as “Modified Kanapilly (phthalate)” in the interlab comparison of MMVF in different pH 4.5 media [19]. It was concluded that “The type C liquid with the phthalate buffer gives results which in most cases are comparable with those obtained with the acidified Gamble's liquid (type B)” [19]. Additionally, observations match: regarding Mg (and Al) leaching at pH4.5 but not at pH7.4, or regarding the ratio of rates obtained at different SA/V ratios.
3. de Meringo et al. measured k for SA/V ranging from 10 to 400 h/cm, which extends farther than our SA/V range [21, 28]. Our data confirms that higher k can be observed at lower SA/V, and our actual acceleration is consistent with factors observed in their study (Table 3), but our average SA/V of 83 h/cm (inversely, V/SA = 0.033 μm/s) is fully consistent with earlier data. E.g., Guldberg et al. specify V/SA = 0.030 μm/s (corresponding to SA/V = 92 h/cm, fully consistent with our screening) [14, 19]. The BIA report even recommends “a low [V/SA] ratio of 0.003 μm/s proved to be the most favorable condition to relate to in-vivo data” [18].
4. Concerning ion analysis, we follow the advice from Guldberg et al. to calculate our k values based on dissolved ions during 25-30 days [19] (32 days in our case). They recommend Si, optionally Al. We follow Potter to add the oxides of Si and Al for k determination, [27] and additionally report kinetics of Mg to assess leaching. We did not differentiate initial k vs. average k, as proposed by initial studies of de Meringo [21]. The limited accuracy of BET determination imposes an uncertainty of up to 30% for the conversion from measured ions to k values. However, BET is not required to compare ion dissolution as-marketed vs. binder-removed, or pH4.5 vs. pH7.4, or entire vs. respirable-only.
5. Finally our sampling concept is fully consistent with the Guldberg et al. method, [14] but adds more. We believe this to be an improvement of reliability, as the between-sampling volume represents more than 90% of all ions, whereas earlier concepts only analyzed the samples, which hold about 10% of all ions. It allows us to cross-check the mass balance between remaining solids (gravimetric) vs. interpolated ion samplings vs. cumulative ion sampling. We find excellent consistency between the two ion-based methods (Additional file 1: Figure SI_6b), and very good consistency with few % mismatch between either of the ion methods and the remaining solids (Additional file 1: Figure SI_6a). One outlier of significantly lower remaining solids (64%) than expected from 19% dissolved Si, Al ions (MMVF #4, thermal binder removal), may be related to additional leaching effects, as observed on Mg (Figure 5a), but may also indicate that because this is the only case of dissolution >> 10% a more complicated calculation would be beneficial. Thelohan and de Meringo support that this is not required for low overall dissolution [28]. On average across the entire test set, the mass balance is 100.1 ± 4 % (min 95%, max 110%), which we take as strong support for the validity of the unexpectedly low dissolution rates.

In summary, we believe our dissolution methodology to be valid and appropriate for comparison against literature data. Thus, the reasons identified for the slow dissolution rates are the oxide composition (discussed above) and the presence of the binder (discussed below).

### Binder

We agree that studies without binder are highly relevant for mechanistic understanding of shape-induced efffects (the “fibre paradigm”), but not for assessment of occupational hazards. Studies without binder do not address an occupational scenario (such as a traded intermediate), but performed a post-processing of the as-marketed MMVF to remove the binder or tested non-commercial materials [29]. In reality, modern stone wool MMVF were found to be coated by 2.8 ± 1.0 % binder (Table 1). This value is consistent with literature [2]. Our nitrogen adsorption isotherms show that the binder reduces the adsorption energy of the first nitrogen layer significantly across the ensemble fibre surface, not only locally. The reduction of specific surface area by the binder covering fibre-fibre touching points is moderate and does not suffice to explain the reduced release of ions in the presence of the binder.

By optical microscopy of one example of glass wool MMVF, Potter and Olang found no change in diameter shrinkage rates in pH 7.4 with or without a novel carbohydrate-polycarboxylic binder [30]. Due to the significant differences in the composition of glass wool and binder (in their example: 67.9 % SiO_2_, 1.3% Al_2_O_3_ with droplets of a *hydrophilic* binder that “swells in water”), extrapolation to stone wool MMVF coated by *hydrophobic* binder and oil is impossible. To the best of our knowledge, the effect of binder coatings on stone wool MMVF has not been reported. Our direct comparison of dissolution kinetics (Fig. 5, Table 3) evidences a significant acceleration of stone wool dissolution by removal of binder by either of the two removal processes tested. Especially leaching of Mg is suppressed in the presence of the binder (Fig. 5). This can be a direct effect of the binder layer or an indirect effect via a silica-rich gel layer. The occurence of gel on dissolving MMVF was observed already in the 1984 WHO proceedings, and its slowing effect on dissolution was discussed in detail [1]. Guldberg et al. highlighted that in their dissolution studies on high-alumina-low-silica MMVF –tested without binder– gel formation was reduced, and they specifically attributed this to the increase in Al/(Al + Si) ratio [16]. It is intriguing to compare our SEM results in Fig. 3 and 4: If we remove the binder, we reproduce the observation of Guldberg et al., as the surface of our binder-removed MMVF after dissolution testing is smooth, does not show leaching pits, and no obvious gel layer. This holds for both processes that we tested to remove binder, and for both MMVF#4 and MMVF#7 (Fig. 3 and 4). In contrast, the same materials with their binder show a pronounced gel formation (Fig. 3 and 4). The gel formation with leaching pit features was actually observed for the entire test set of modern MMVF, tested with binder (Additional file 1: Figure SI_5 summarized in Table 3. Even more SEM results are documented in Additional file 2: SEM Annex). Potentially the binder-induced gel formation is a mechanism contributing to the dissolution rates (tested with binder) being lower than in previous literature (tested without binder). Highly resolving SEM scans were unfortunately not reported for the one example of glass wool dissolution with hydrophilic binder [30].

### Classification based on composition and biopersistence

The reduction of dissolution for the specific composition (and binder) is relevant, as the range of k we measure is the borderline region correlated by IARC to the change of pathogenicity from MMVF21 and MMVF34 in terms of their fibrogenic potential (Table 4): MMVF21 caused pulmonary fibrosis, but MMVF34 did not. In 107 rats exposed to MMVF34, no carcinoma and five adenomas were observed. In the 107 rats in the control group, one carcinoma and three adenomas were found [10]. All the modern MMVF tested here where significantly different in their composition from MMVF34, which actually is an extreme point already in the “biosoluble MMVF” CAS compositional range (with highest Al/(Al+Si)). MMVF34, tested without binder, also had the highest biosolubility within the CAS range [16]. Only for MMVF34, tested without binder, the absence of chronic inhalation effects [17] and absence of cancerogenicity [15] were reported. Thus, exoneration of the CAS range was a) extrapolated from materials without binder and b) extrapolated from MMVF34 motivated by dissolution tests [16]. Table 4 summarizes our results on composition and dissolution, and benchmarks modern MMVF against the IARC / CAS ranges and representative MMVF.

**Fig. 6:**
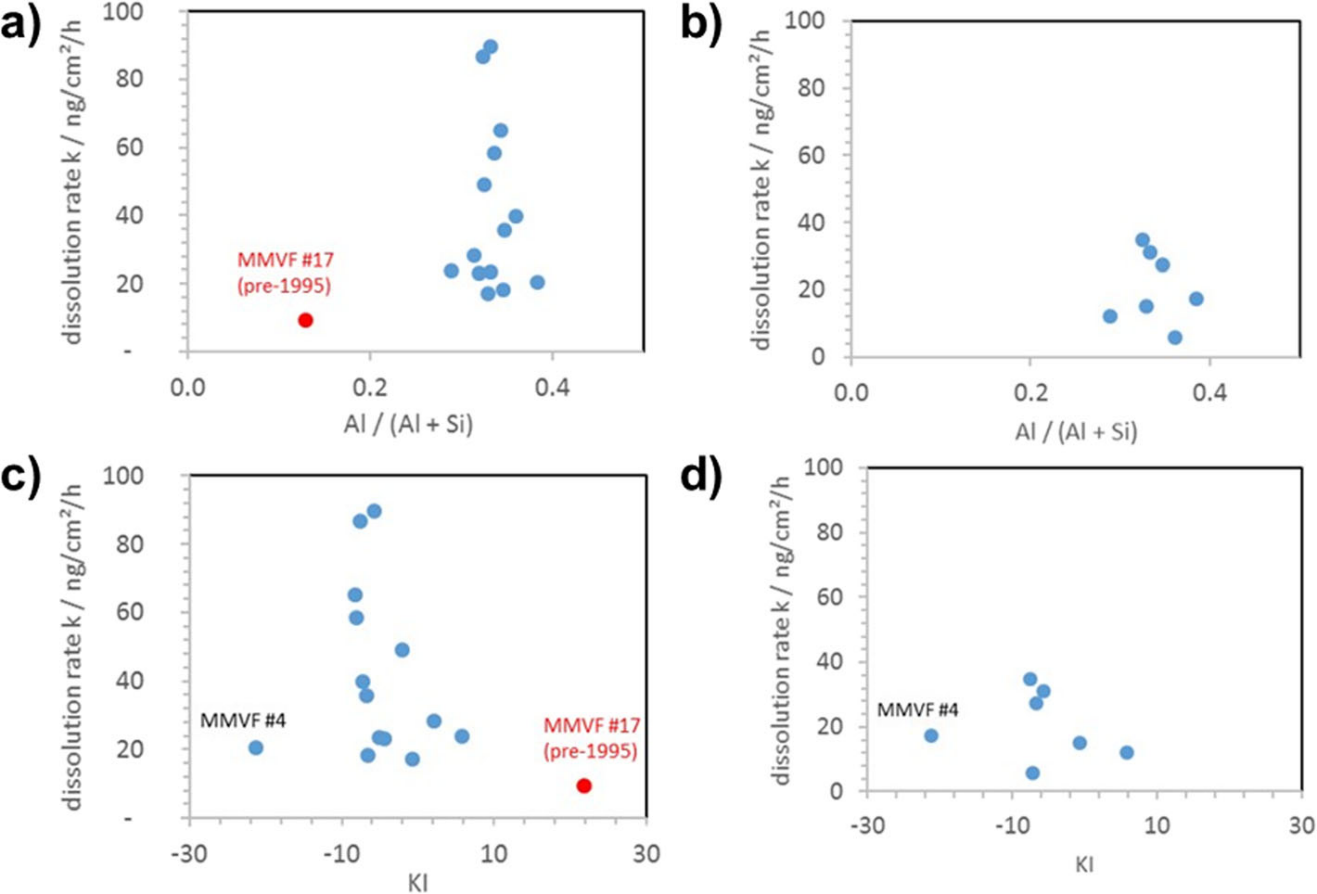
Summary of measured dissolution rates k [Si,Al, ng/cm^2^/h] as a function of the compositional parameters **a**,**b** of the molar ratio Al / (Al + Si) or **c**,**d** of KI. The flow rates are identical throughout, and the pH is **a**,**c** pH 4.5 and **b**,**d** pH 7.4. The materials with highest k at pH 4.5 (MMVF #1, MMVF #5) were also screened at pH 7.4 and are included in the plot

For visualization, Fig. 6 plots the k results in the Al/(Al+Si) metrics. No trend can be identified because of the low spread of the modern MMVF in Al/(Al+Si). As an alternative visualization, the k results are plotted in KI metrics. KI is defined by obsolete German regulation, now overruled by CLP, as the sum of content of the oxides of Na, K, B, Ca, Mg, Ba minus twice the content of Al oxide [31]. Interestingly, KI separates MMVF #4 and MMVF #17 apart from the other MVMF, and a trend of k in the KI metric is suggested by the available results, but overall KI is not helpful to predict k. The differentiation of MMVF #4 is expected because it is an innovative product combining the high temperature performance of stone wool with the thermal, acoustic and low weight benefits of glass wool. This new type of mineral wool with a composition similar to that of stone wool, processed through a high temperature version of glass wool fiberising spinner, does show the same binder-induced gel formation as our other test materials, but reduced sensitivity on pH (Table 3). Regardless, we do not aim to establish any new predictive parameterization of composition. Instead, the composition analysis simply shows that modern MMVF do not all fall into the CAS range of exonerated MMVF. *Independently*, the dissolution rates are found to be lower (average 41 ng/cm^2^/h) than expected for exonerated fibres that are biosoluble with no effects in intraperitoneal injection (ip) cancerogenicity studies and in chronic inhalation studies (>400 ng/cm^2^/h, Table 4). The most important systematic uncertainty of 30% on absolute k values is our use of BET for surface area determination, but that does not compromise significance against the exonerated fibre values. Only the in vivo studies are relevant for current MMVF classification [10, 32], but biosolubility is decisive to prevent fibre-induced pathogenicity [6, 33, 34]. Specifically for MMVF, in vitro dissolution testing is known to correlate well with biosolubility and ultimately with pathogenicity [10, 15–17, 26]. Classification relies on in vivo clearance rates and/or fibrogenic and/or carcinogenic potential [9, 32]. Our present contribution hence remains a screening. We emphasize the importance of validating the present findings by appropriately designed in vivo studies that also use high resolution counting methodology to determine the dimensions of retained fibers with and without binder in the lung.

### Potential of exposure

To complement the hazard screening, the potential exposure needs to be known to assess the urgency of action. According to the European Standard EN 481 “Size Fraction Definition for Measurement of Airborne Particles” (1993), the thoracic fraction is that portion of the inhalable particles that pass the larynx and penetrate into the conducting airways (trachea, bifurcations) and the bronchial region of the lung (D50 = 10 μm), whereas the respirable fraction is the portion of inhalable particles that enter the deepest part of the lung, the nonciliated alveoli (D50 = 4 μm). Thus, our studies (Table 2) show that the respirable fraction of modern MMVF, assessed here by a 4 μm cutoff, is not less than in pre-1995 MMVF. The function of the binder is obviously to bind the fibres together, and thus it is beneficial to reduce exposure, but the IARC collected evidence that both manufacturing at MMVF production plants and installation at construction sites generates respirable airborne dust containing WHO fibres in a wide range approximately from 0.01 to 1 fibres/cm^3^ [10]. Overall, relevant exposure cannot be excluded.

## Conclusion

The compositional range of modern MMVF products (Table 4 and the additional products in Additional file 1: Table SI_1), is not compatible with the reference material MMVF34, that was used as benchmark to assess fibres with high-alumina, low-silica compositions, which consequently were exonerated from carcinogen classification. Instead, the compositional range of modern stone wool extends between MMVF34 (biosoluble stone wool) and MMVF21 (low biosolubility stone wool) reference materials as limiting cases, with a compositional average matching quite exactly the midpoint between MMVF34 and MMVF21. These results are not limited to a single producer or to a single country of origin, but cover 5 producers from 6 countries. The dissolution rates at pH 4.5, measured by a replicate of setups that were developed and validated in the 1990ies, are an order of magnitude slower than those reported for biosoluble MMVF34. This is significant considering all known sources of error. Despite the SiO_2_ content of an average modern MMVF being reduced vs. the historical benchmark MMVF21, the measured average dissolution rates at both pH 7.4 and pH 4.5 are within 20% identical to MMVF21 (Table 4). This is explained, at least in part, by the presence of up to 4% binder that coats the MMVF and has a significant influence on dissolution, probably by favoring gel layer formation. Here we tested MMVF as they are marketed and handled, i.e. with binder, whereas it appears that previous hazard assessment relied on abiotic, in-vitro and in-vivo studies with MMVF dissolution accelerated by deliberate removal of the binder. Considering that modern MMVF, with their binder, have actual dissolution rates ranging from 6 ng/cm^2^/h to 171 ng/cm^2^/h, which is the borderline range correlated to the onset of lung fibrosis and thoracic tumors, [10] and considering further the content of respirable fibres, the risk assessment of modern stone wool may need to be revisited. However, in vitro dissolution studies remain indicative and cannot replace nor predict in-vivo studies of MMFV as marketed (with binder).

## Additional files

Additional file 1: Online Supporting Information: Schematics of respirable fibre fractionation and of dissolution testing. Additional results on MMVF composition, on respirable fibre content and on dissolution kinetics at pH4.5. Characteristic features in SEM scans before and after dissolution testing. Cross-correlation of three metrics of dissolution analysis. (PDF 967 kb)

Additional file 2: Annex: extensive SEM before and after dissolution testing. (PDF 8398 kb)

## Footnotes

1 “Es ist weiterhin notwendig, nicht nur binderfreies Material zu untersuchen, das in der Arbeitswelt kaum auftaucht, sondern Fasern in handelsüblicher Form, d.h. mit Bindermaterial.“ (BIA report 02/98, Conclusions p. 139, endorsed also by “Stellungnahme R.C. Brown, U. Nebe, ECFIA: …Im übrigen teilen wir die Ansicht, daß es weiterhin notwendig ist, nicht nur binderfreies Material zu untersuchen, das in der Arbeitswelt kaum auftaucht, sondern Faserprodukte in handelsüblicher Form.“ p. 279). Author’s translations: “Furthermore it is necessary to investigate not only binder-free material, which is uncommon in the working environment, but fibres in the form that is customary in trade, i.e. with binder material.“ and “…by the way we share the view that it is necessary to investigate not only binder-free material, which is uncommon in the working environment, but fibre products in the form that is customary in trade,“
