# Supplementary material for: Composition, Respirable Fraction and Dissolution Rate of 24 Stone Wool MMVF with their Binder
Source: Part Fibre Toxicol. 2017 Aug 7;14:29. doi: 10.1186/s12989-017-0210-8 (PMC5547462; doi:10.1186/s12989-017-0210-8)
Supplement: Supplementary file 1 — Online Supporting Information: Schematics of respirable fibre fractionation and of dissolution testing. Additional results on MMVF composition, on respirable fibre content and on dissolution kinetics at pH4.5. Characteristic features in SEM scans before and after dissolution testing. Cross-correlation of three metrics of dissolution analysis. (PDF 967 kb) [file 12989_2017_210_MOESM1_ESM.pdf]

Composition, Respirable Fraction and Dissolution Rate of 24 Stone Wool MMVF with their Binder

Wendel Wohlleben*, Hubert Waindok, Björn Daumann, Kai Werle, Melanie Drum, Heiko Egenolf

Supporting Information

Method of MMVF composition analysis for Boron:

Sample pretreatment for B:

Approx. 100 mg of the sample was weighed, to the nearest 0.1 mg, into a glass carbon crucible. 2.5 ml of caustic soda solution (ß = 400 g/l) were added, and then evaporated to dryness on a hot plate. The residue was then treated with a fan burner until a clear melt was obtained. After cooling down, the melt cake was dissolved in 30 ml of deionised water, and 15 ml of semiconc. hydrochloric acid were added. The solution was filled up to a total volume of 100 ml with deionised water.

Measurement of B:

Boron was determined by inductively coupled plasma-optical emission spectrometry (ICP-OES). Prior to taking the measurement, the instrument was optimized in accordance with the manufacturer's specification.

Apparatus:	ICP-OES Spectro Arcos	
Parameters:	Wavelength [nm]:	B 249.773
	Dilution factor:	1
	Nebulizer:	Meinhard 1 ml
	Nebulizer flow:	0.75 l/min
	Pump rate:	15 rpm

Measurement:	3 replicates
Integration Time:	48 s
Calibration:	external; ß = 0 / 10 mg/l (matrix-matched standards)

Method of MMVF composition analysis for K, Na:

Sample pretreatment

Approx. 150 mg of the sample was weighed, to the nearest 0.1 mg, into a platinum dish and 10 ml of a mixture of H2SO4 conc. and deionised water (volume ratio 1:4) and 10 ml hydrofluoric acid (40 %) was added. The platinum dish was placed on a sand bath and boiled down to dryness. After cooling down the platinum dish, the residue was dissolved in deionised water by careful heating, and 5 ml of semi-conc. hydrochloric acid were added. Finally, the solution was filled up to a volume of 100 ml with deionised water.

Measurement of K, Na:

The determination of K and Na in the sample solution was carried out by Flame Atomic Absorption Spectrometry (F-AAS). Prior to taking the measurement, the instrument was optimized in accordance with the manufacturer's specification.

Apparatus:	F-AAS Shimadzu AA-7000	
Parameters:	Wavelengths [nm]:	K 766.5 Na 589.0
	Gas:	Air/acetylene
	Slit width:	0.7 nm (K) / 0.2 nm (Na)
	Calibration:	external (matrix-matched standards)

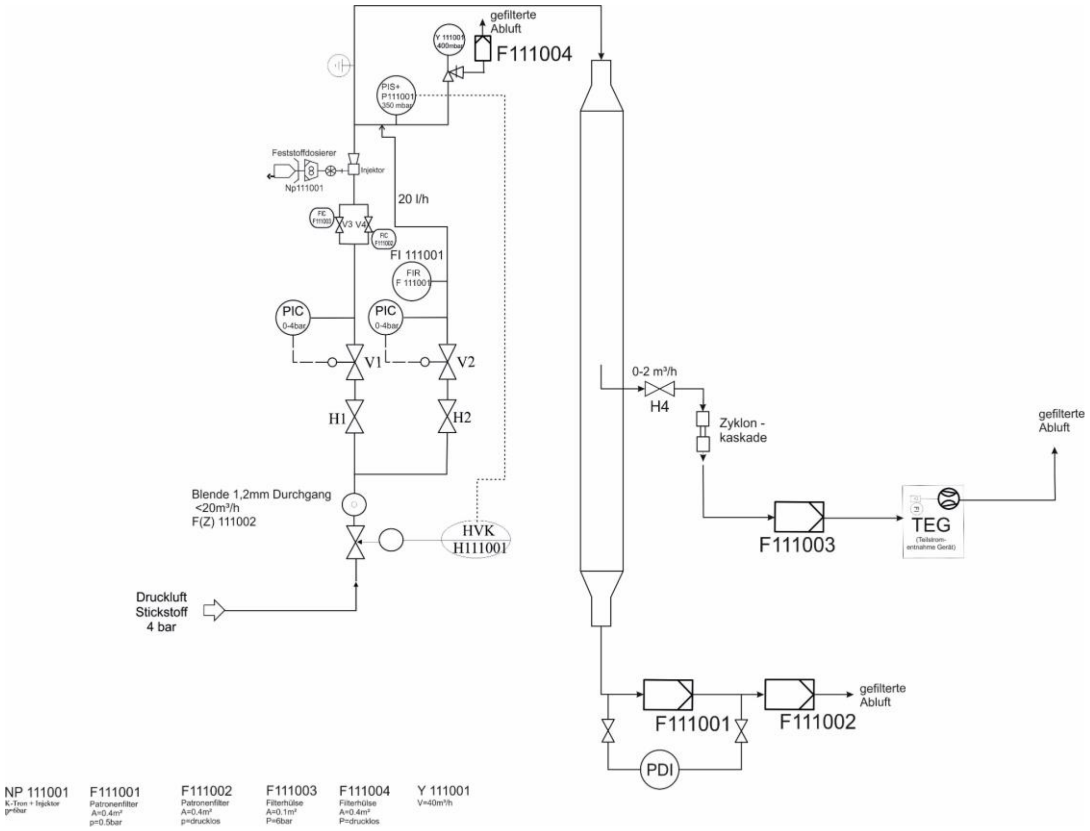

Figure SI 1 Aerosol fractionation setup schematic

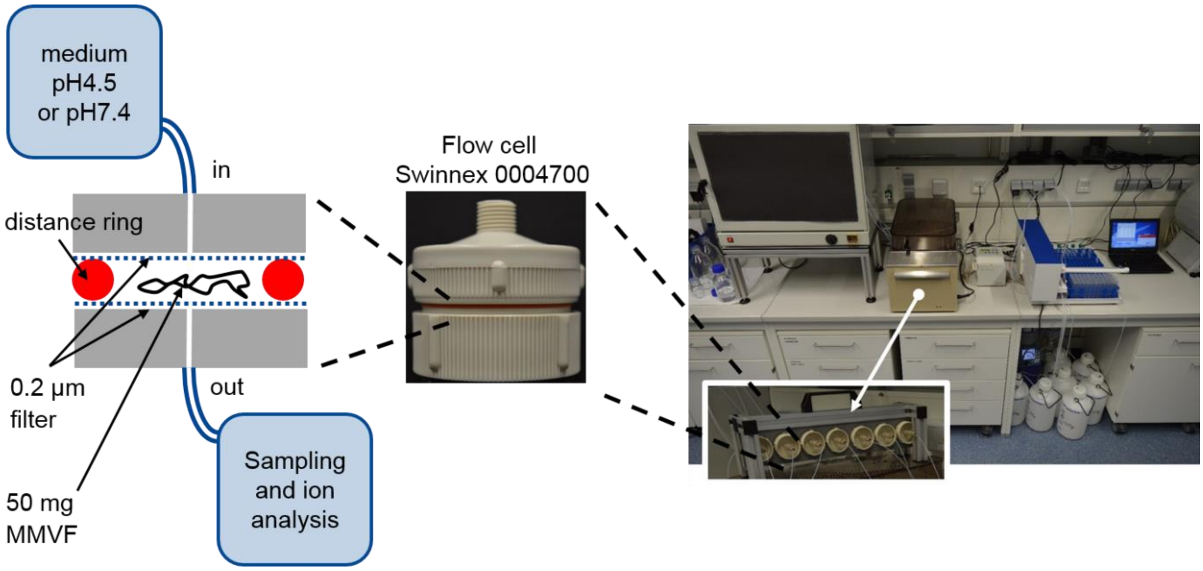

Figure SI 2 Dissolution testing: schematic of the dynamic (flow-through) system setup and photograph. Photo, from left to right: Thermostatted & stirred dissolution medium (VWR „Enghals-Kanister“, Art.no. 216-5376, 25 liters), from where pre-filters (UHMWPE) ensure that the medium is free of particulates before entering the sample cells. Seven parallel PEEK tubes feed seven parallel

dissolution cells (see inset), which are temperature-stabilized at 37°C in a thermostat (Julabo SW-20C) in the middle of the photograph. The peristaltic pump controls the volume flow, and is initialized by the programmable sampler (for the 5 time-resolved cells). Under the table, 5-l-vessels (Kautex, art.no. 350 5L 84131, HDPE) collect eluted medium between the sampling times (for the 5 time-resolved cells) respectively all eluted medium (for the 2 additional cells).

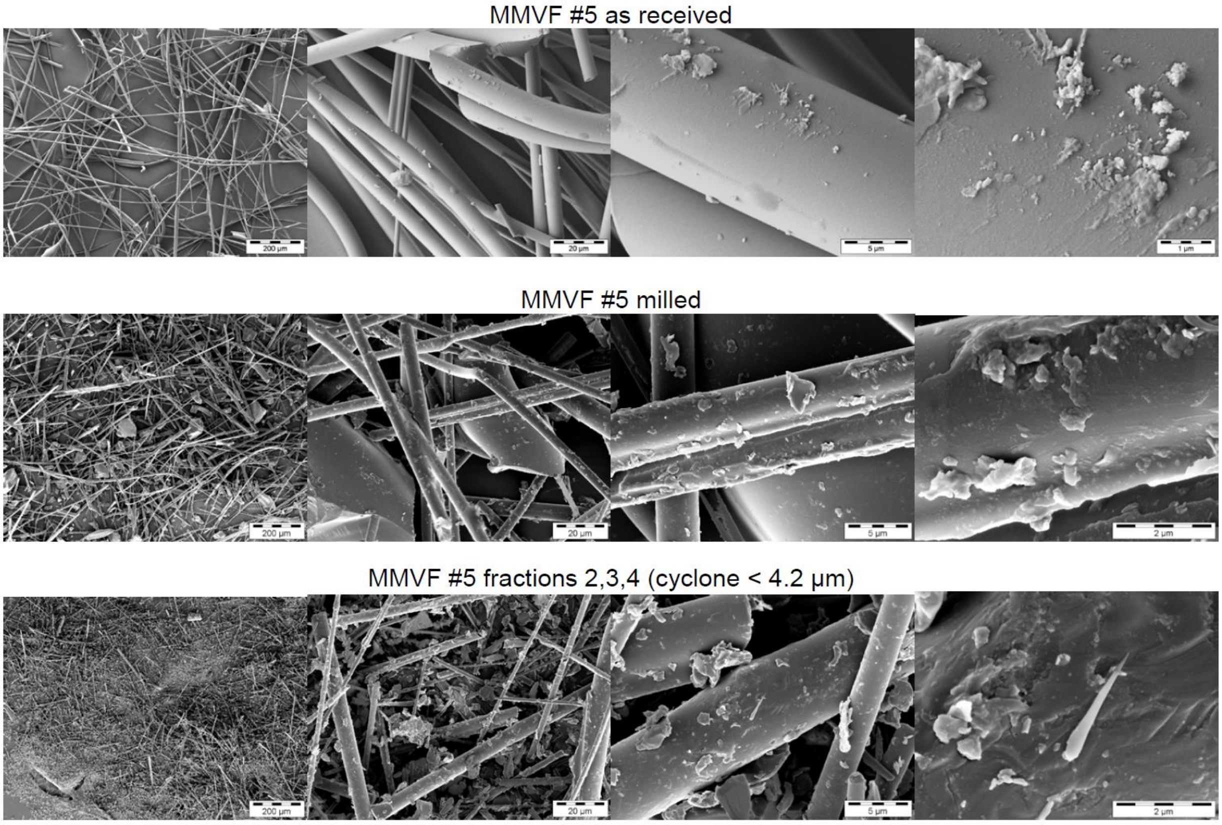

Figure SI_3 SEM micrographs of MMVF #5. As received – after milling – only respirable fraction, all in low – mid – high magnification scans.

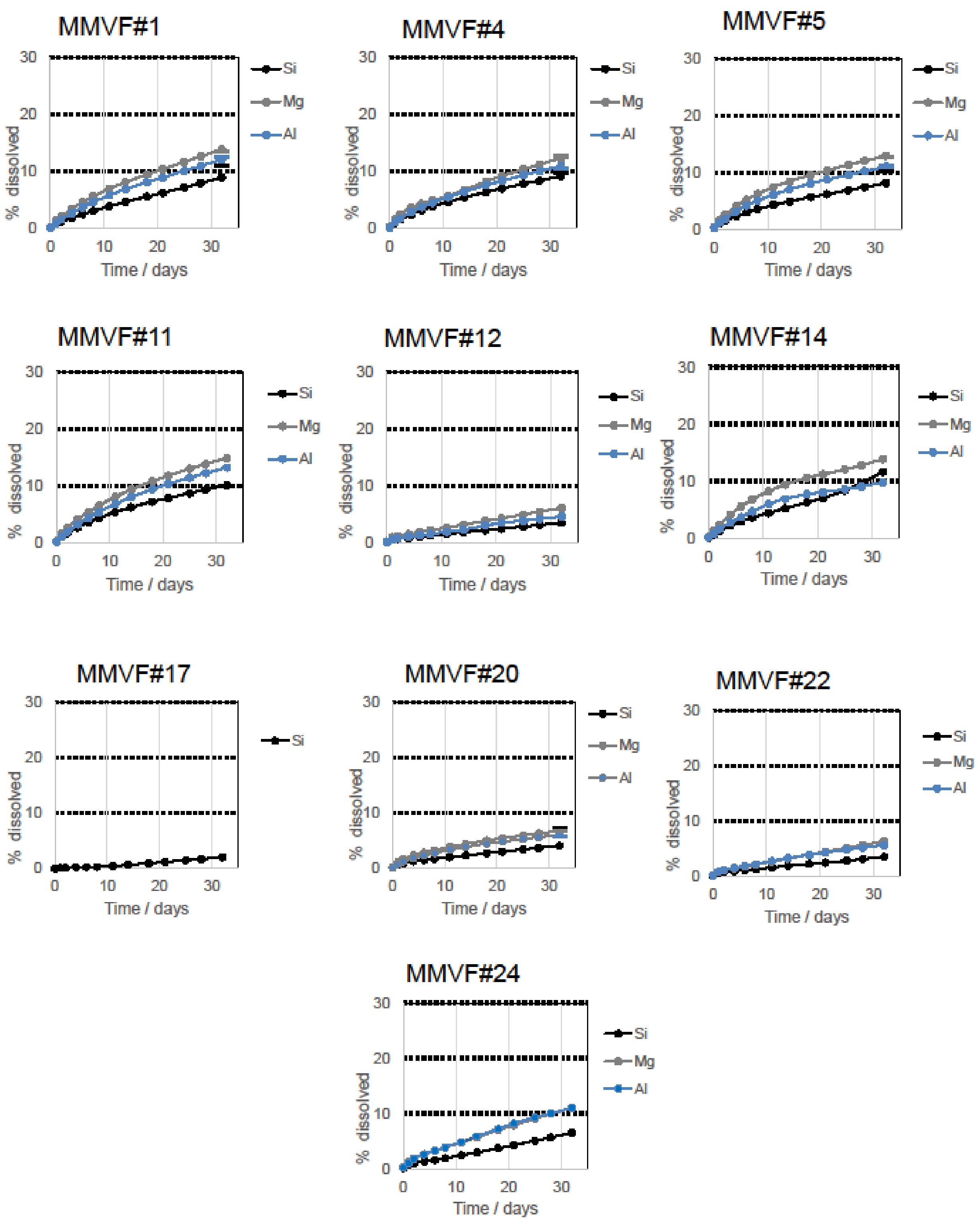

Figure SI_4 Dissolution kinetics at pH4.5 for all materials that were tested with full kinetics. Standard parameters initial mass 50 mg, volume flow 48 ml/d. Percent of oxide dissolved for Si (Black dots + lines), Al (blue dots + lines), Mg (grey dots + lines). For MMVF #17 (pre-1995 material) Al and Mg were below detection limits.

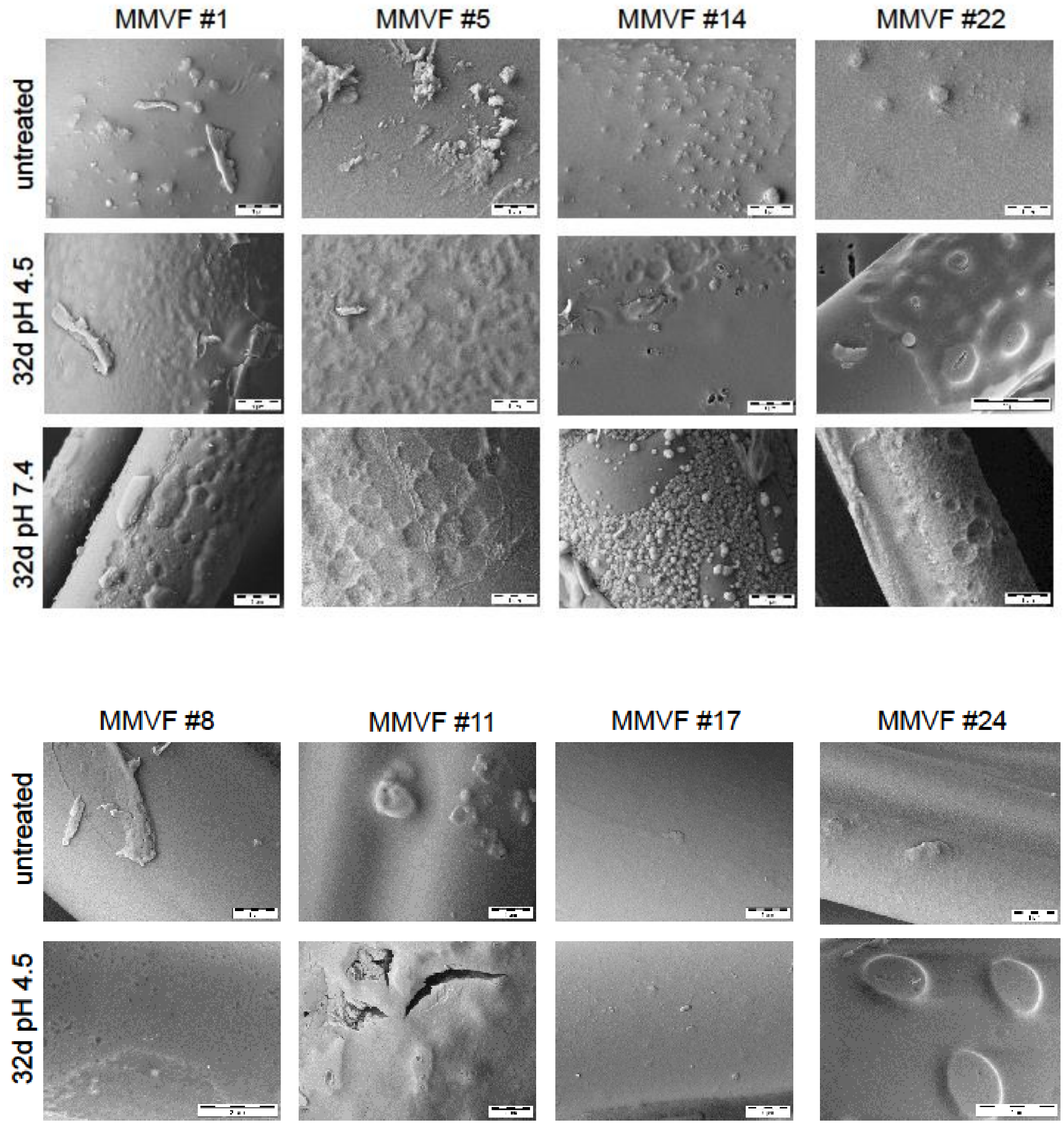

Figure SI_5 Characteristic features of MMVF fiber surfaces before and after dissolution by the conditions indicated (high magnification SEM micrographs). See **SEM Annex** for full documentation of all MMVF with low-mid-high magnification scans before and after dissolution.

a) b)

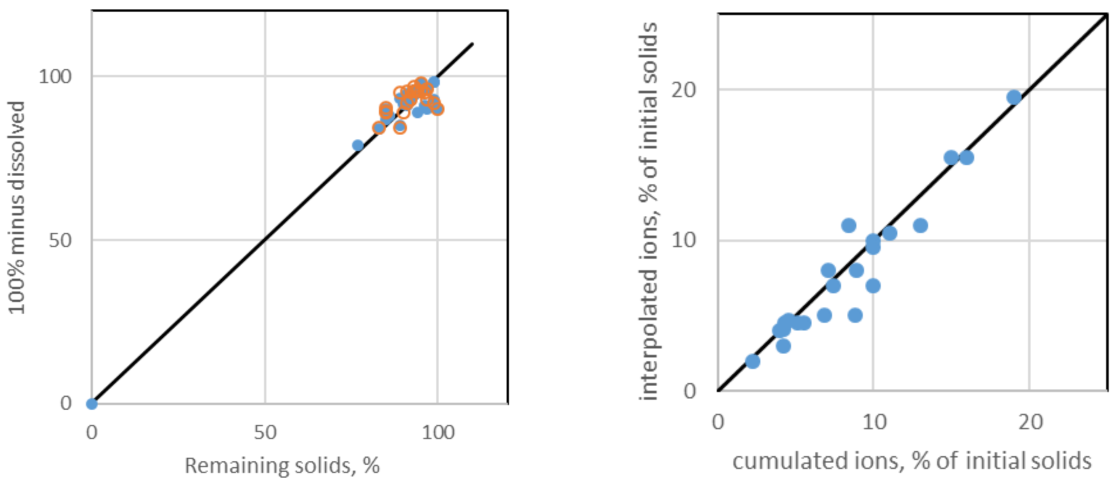

Figure SI_6 Cross-correlation of three metrics of MMVF dissolution: a) remaining solids vs. interpolated ions (based on sampled volume only, which is about 10% of eluted medium, orange circles) and vs. cumulated ions (based on 100% of eluted volume, blue dots). b) interpolated ions vs. cumulated ions. This analysis based on all datasets that were measured with full kinetics (all MMVF treatments with/without binder, pH4.5, pH7.4, all SA/V conditions), only one outlier removed (MMVF #4, thermal binder removal).

Table SI_1 Composition of additional MMVF, for which no dissolution tests were performed.

Country of origin	Producer code	MMVF code	SiO2	Al2O3	CaO	MgO	Fe2O3	TiO2	Na2O	K2O	MnO	P2O5	Cr2O3	BaO	S	SrO	B2O3	SUM	KI-Index	Al / (Al+Si)	BET (m^2/g)	% binder content
Germany	A	MMVF #6	41	19	17	9	7	1.7	1.2	0.6	0.5	0.4	0.2	0.1	0.1	0.1	0.0	97	-9	0.32	0.6	1.2
Germany	A	MMVF #10	40	19	16	10	8	1.2	1.1	1.1	0.9	0.3	0.3	0.1	0.1	0.1	0.0	98	-10	0.32	0.2	3.7
Germany	A	MMVF #13	42	21	17	10	8	1.2	2.2	2.2	1.0	0.3	0.3	0.1	0.1	0.1	0.0	105	-10	0.33	0.7	5.0
Germany	D	MMVF #15	44	16	22	9	7	1.6	2.5	1.6	0.6	0.3	0.1	0.2	0.1	0.1		105	2	0.32		3.6
Germany	A	MMVF #18	41	18	20	8	7	1.7	2.4	1.6	0.5	0.4	0.1	0.1	0.1	0.1	0.0	101	-4	0.31		2.1
Germany	A	MMVF #19	40	18	19	8	7	1.7	2.5	1.6	0.4	0.4	0.0	0.1	0.1	0.1	0.0	100	-5	0.33		4.1
Denmark	A	MMVF #23	41	17	22	10	5	1.7	1.9	0.6	1.1	0.1	0.4	0.1	0.2	0.1	0.0	101	1	0.35	0.3	2.5
Denmark	E	MMVF #25	43	19	20	9	7	0.9	1.8	0.8	0.2	0.7	0.1	0.1	0.4	0.1	0.0	102	-6	0.35	0.3	1.2
Germany	A	MMVF #27	41	19	18	8	8	1.7	2.4	0.8	0.2	0.3	0.1	0.1	0.2	0.1	0.0	100	-8	0.34		2.2
