# Supplementary material for: Composition, Respirable Fraction and Dissolution Rate of 24 Stone Wool MMVF with their Binder
Source: Part Fibre Toxicol. 2017 Aug 7;14:29. doi: 10.1186/s12989-017-0210-8 (PMC5547462; doi:10.1186/s12989-017-0210-8)

SEM - ANNEX

MMVF #1 (untreated reference)

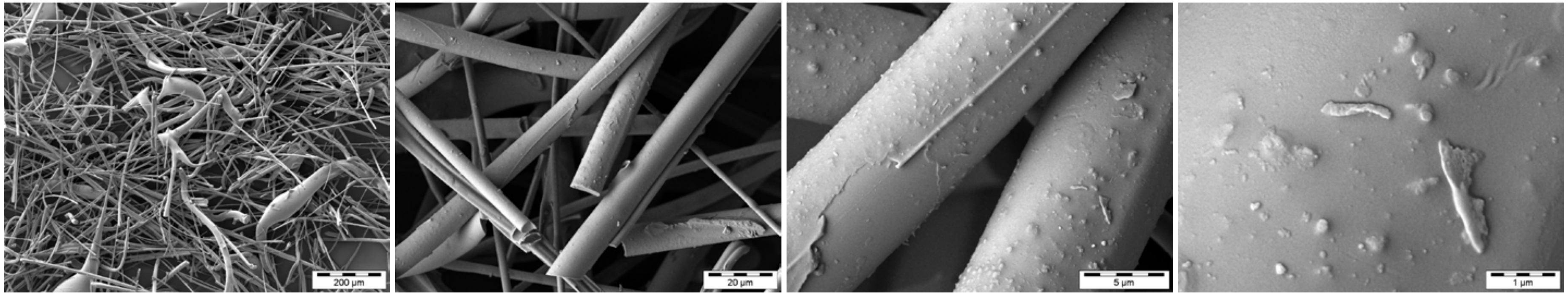

MMVF #1 (32 days pH 4.5)

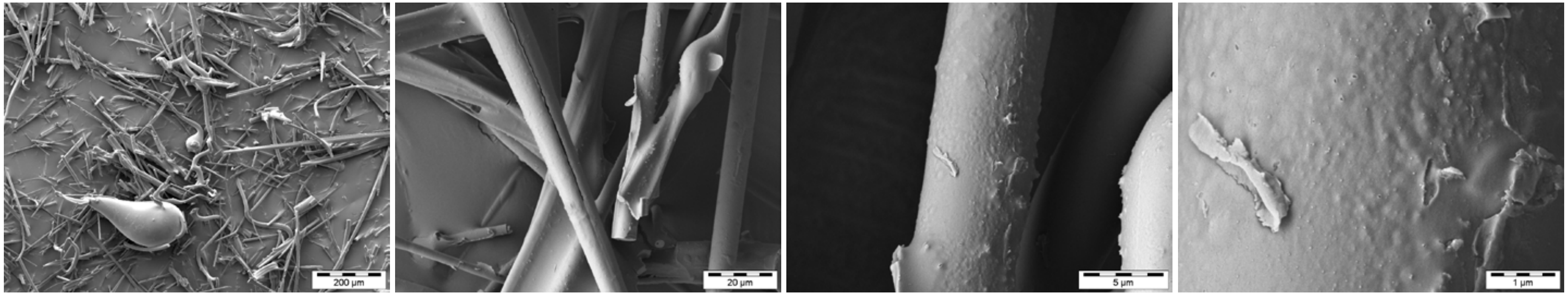

MMVF #1 (32 days pH 7.4)

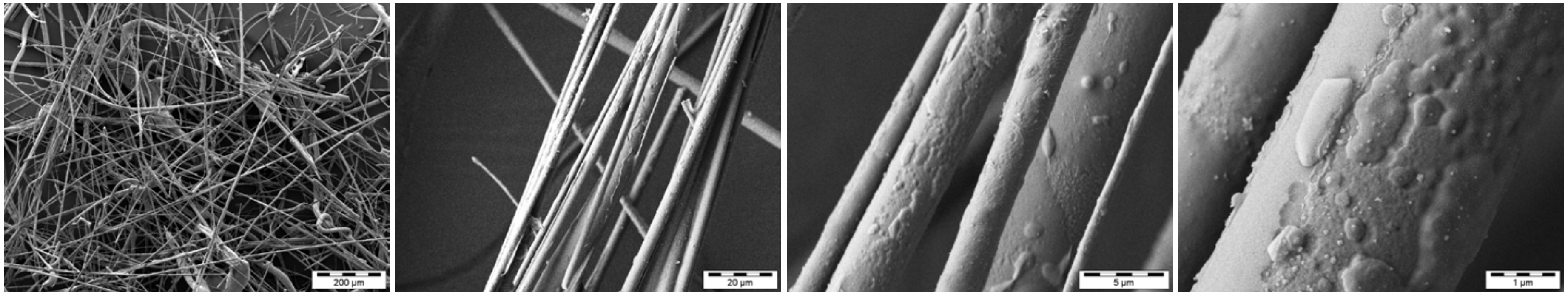

MMVF #2 (untreated reference)

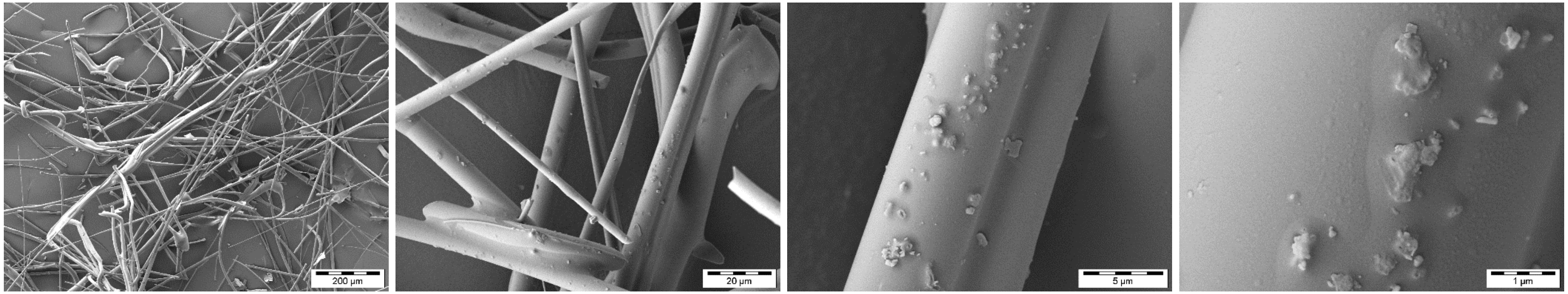

MMVF #4: see manuscript

MMVF #5 (untreated reference)

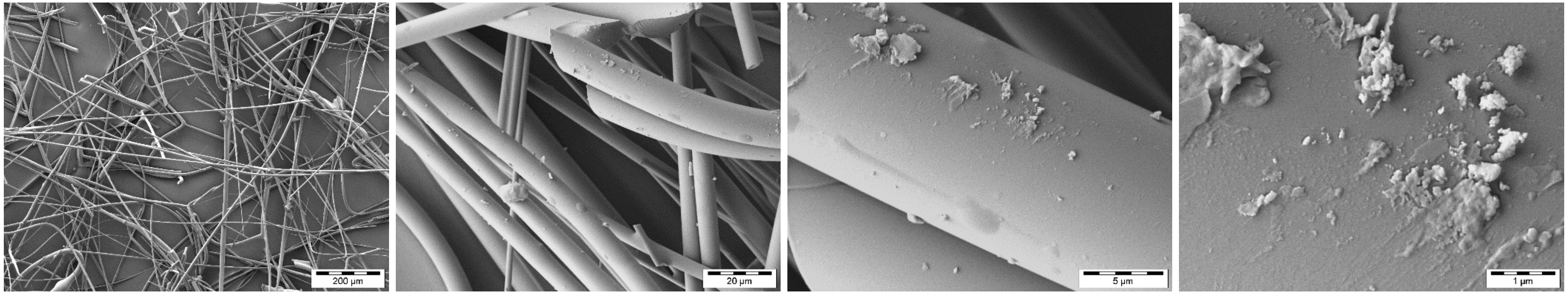

MMVF #5 (32 days pH 4.5)

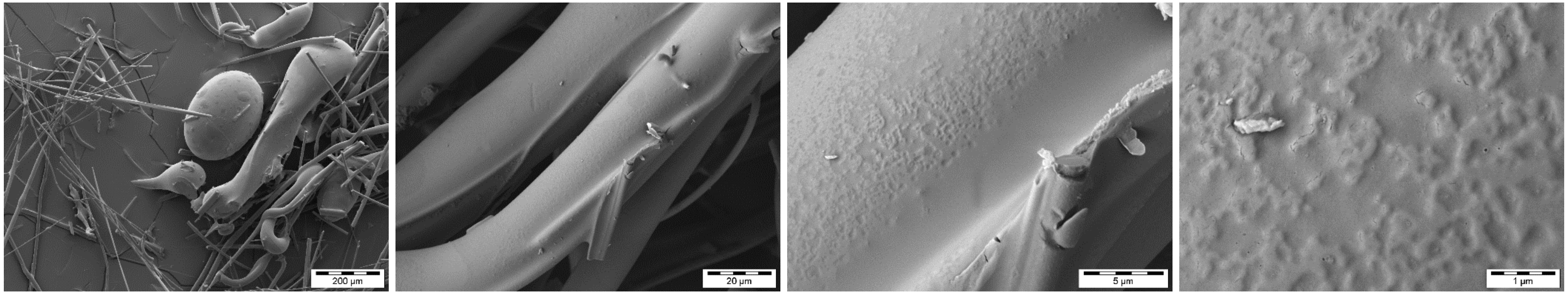

MMVF #5 (32 days pH 7.4)

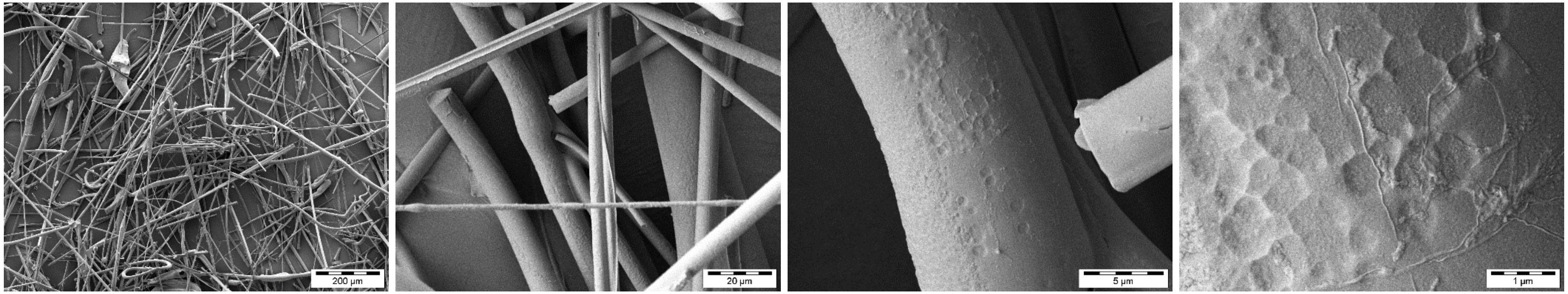

MMVF #5 respirable only (untreated reference)

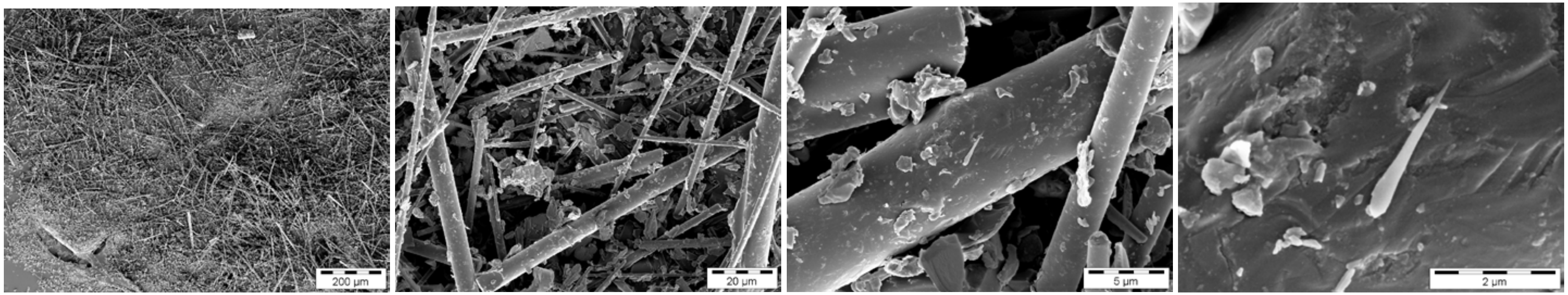

MMVF #5 respirable only (32 days pH 4.5)

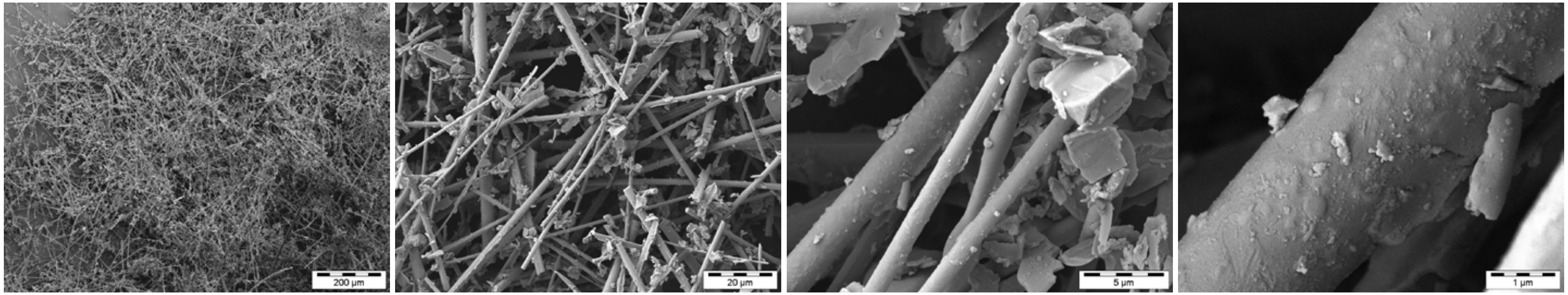

MMVF #7: see manuscript

MMVF #8 (untreated reference)

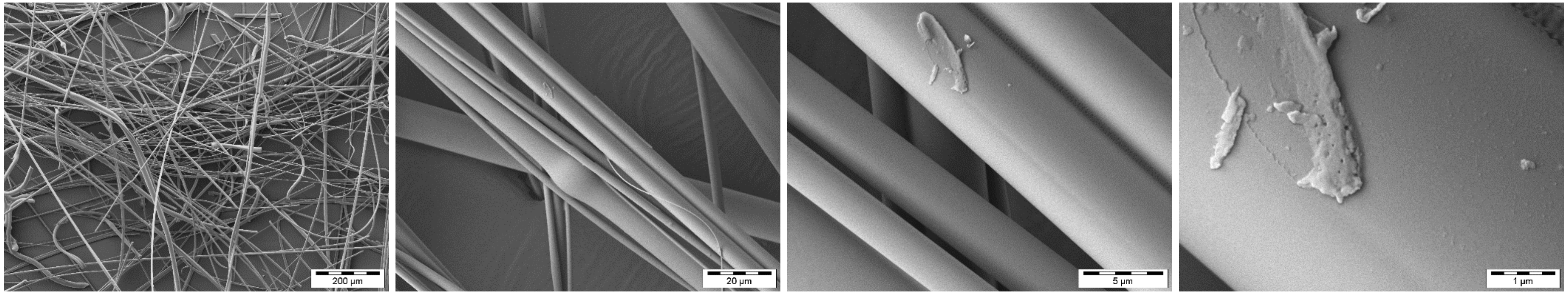

MMVF #8 (32 days pH 4.5)

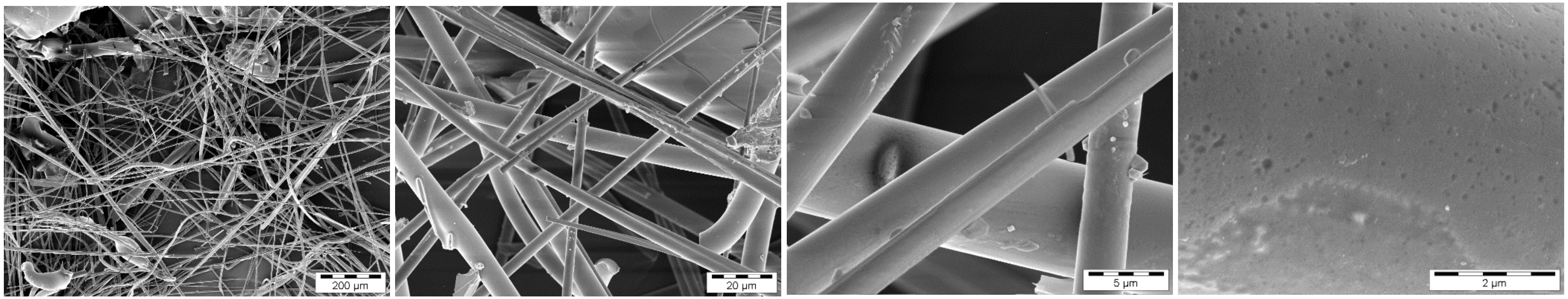

MMVF #11 (untreated reference)

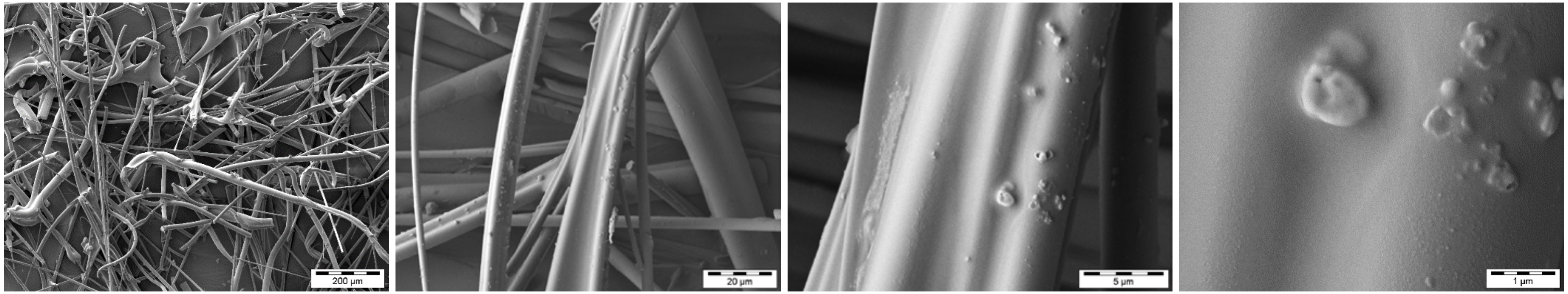

MMVF #11 (32 days pH 4.5)

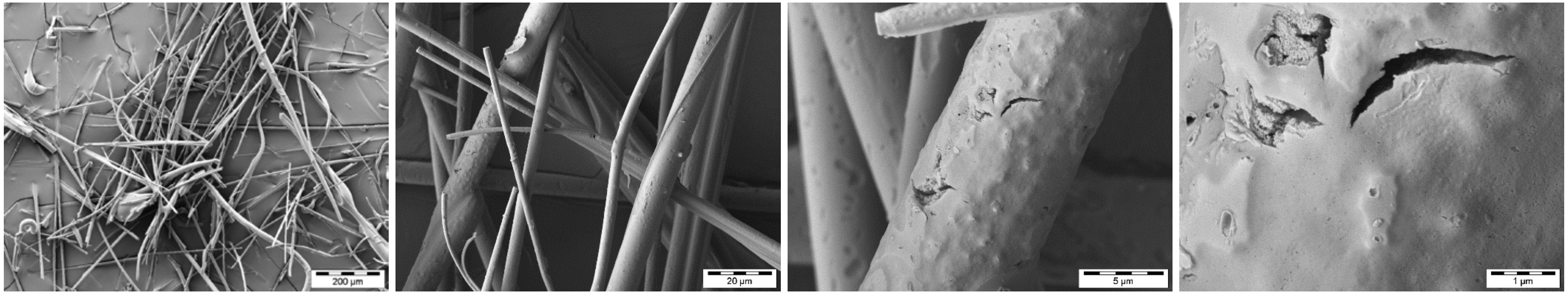

MMVF #12 (untreated reference)

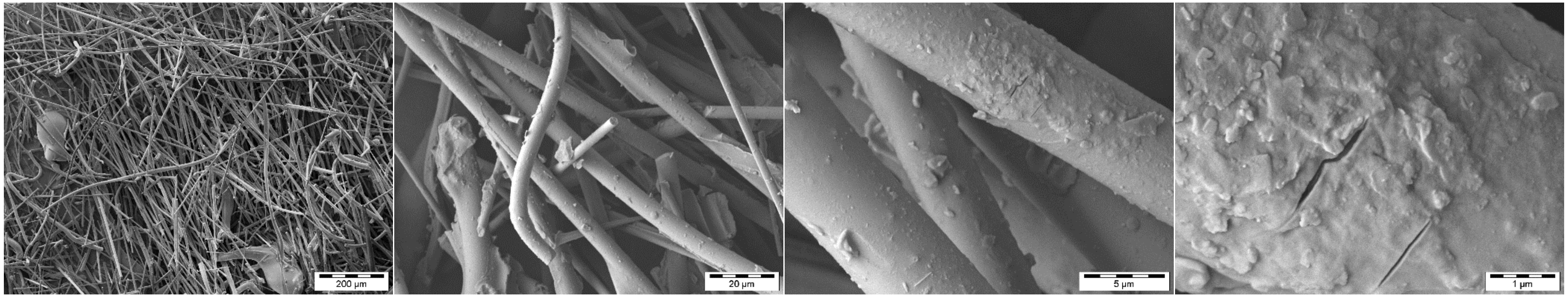

MMVF #12 (32 days pH 4.5)

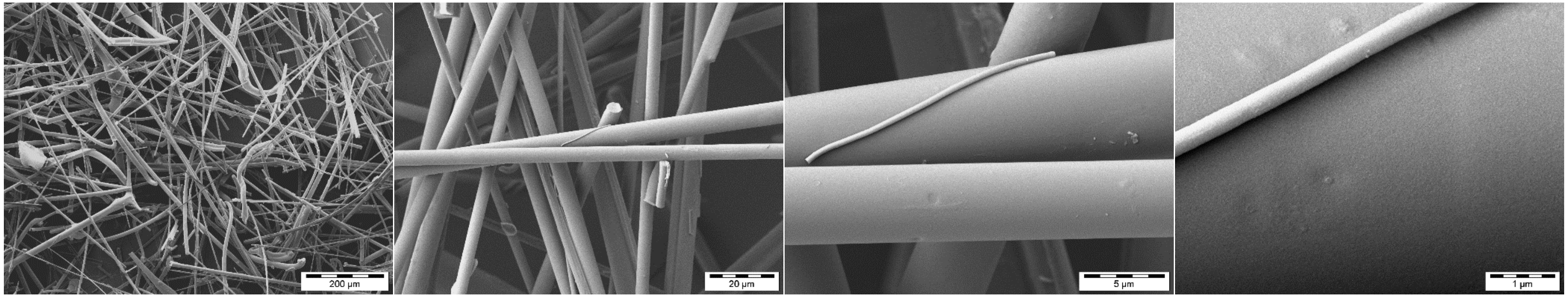

MMVF #12 (32 days pH 7.4)

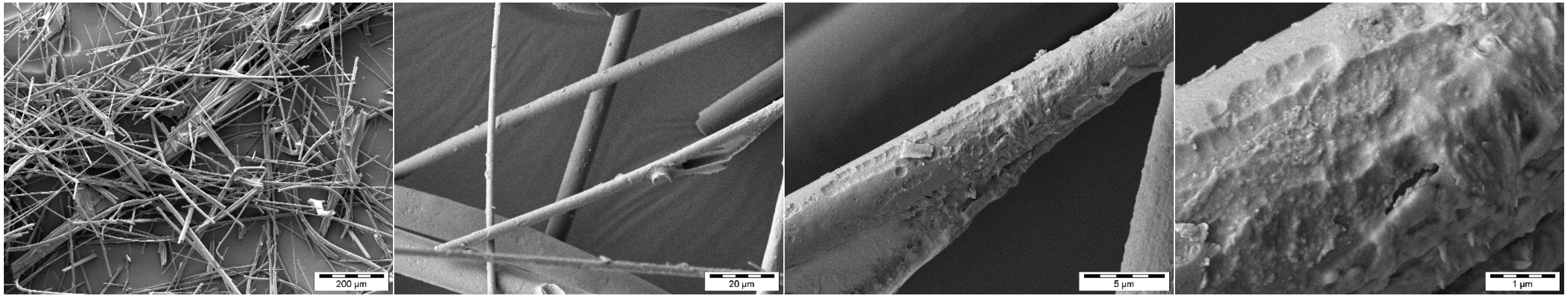

MMVF #14 (untreated reference)

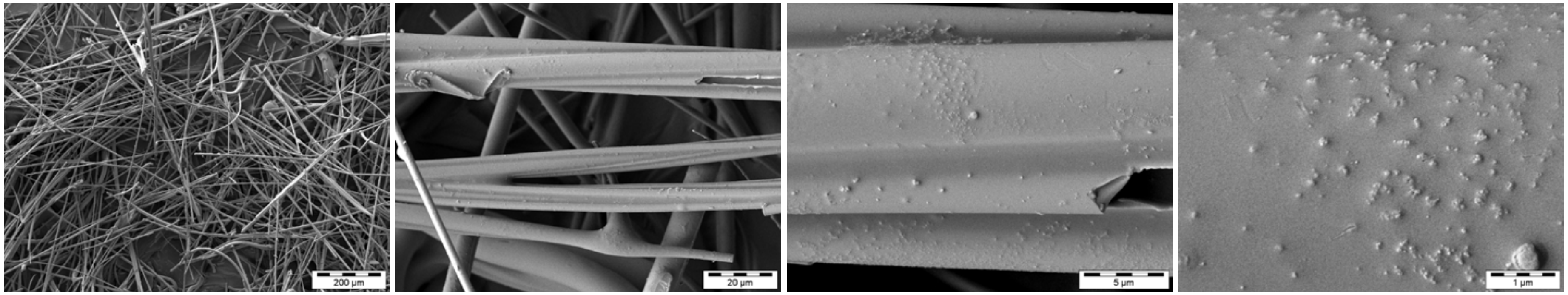

MMVF #14 (32 days pH 4.5)

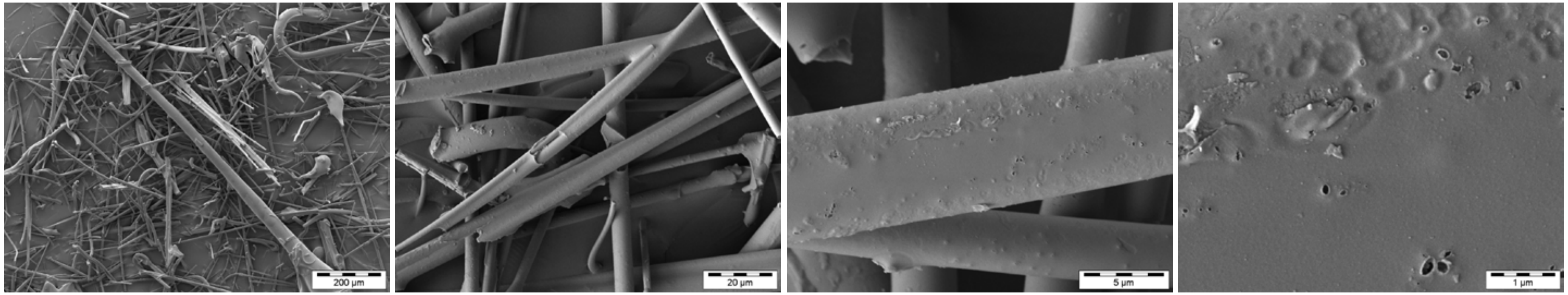

MMVF #14 (32 days pH 7.4)

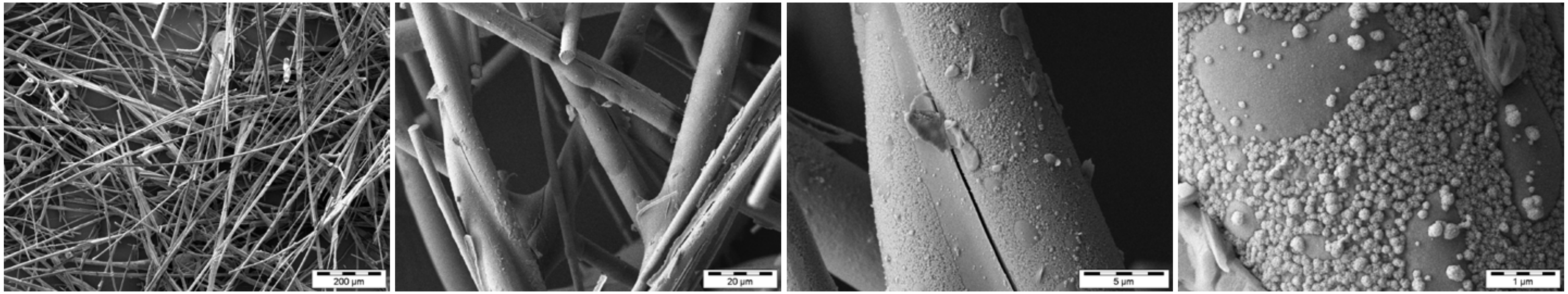

MMVF #17 (untreated reference)

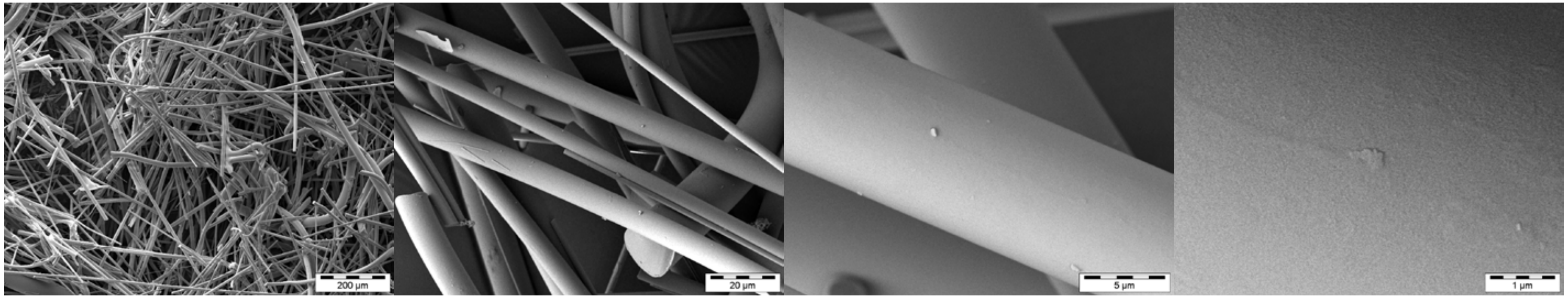

MMVF #17 (32 days pH 4.5)

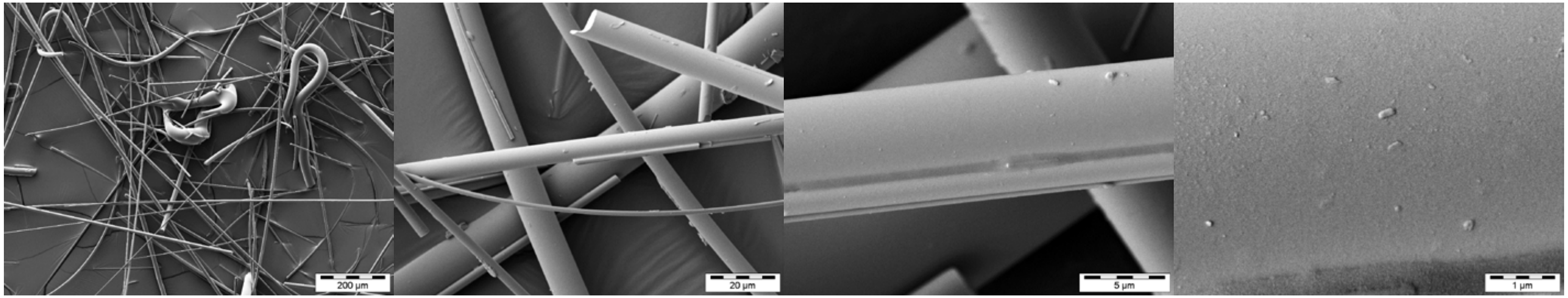

MMVF #20 (untreated reference)

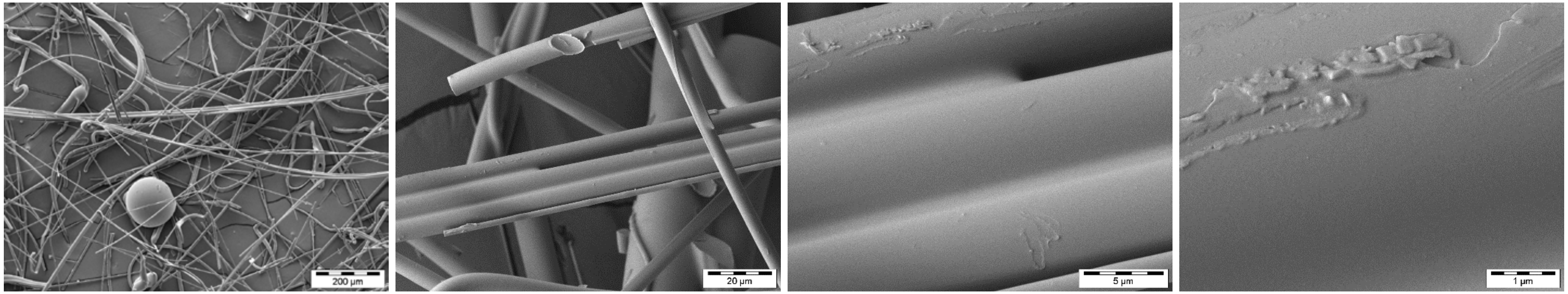

MMVF #20 (32 days pH 4.5)

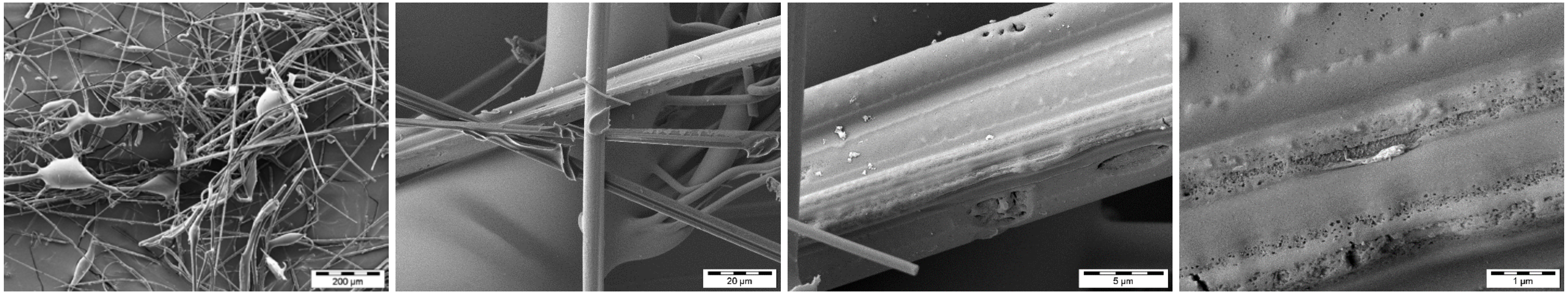

MMVF #21 (untreated reference)

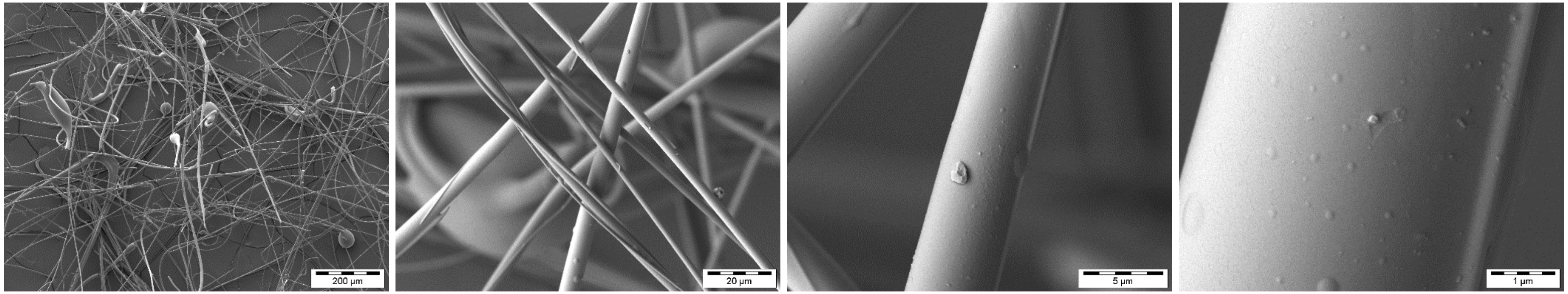

MMVF #22 (untreated reference)

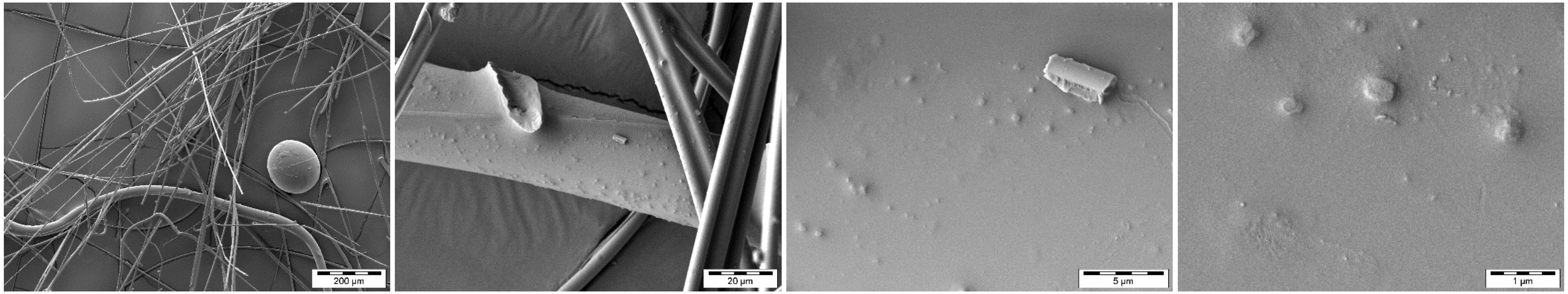

MMVF #22 (32d in pH 4.5)

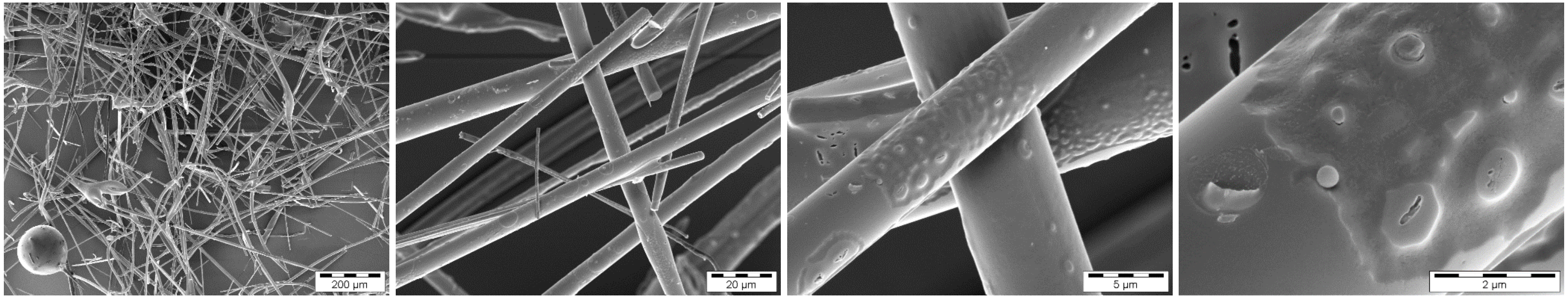

MMVF #22 (32d in pH 7.4)

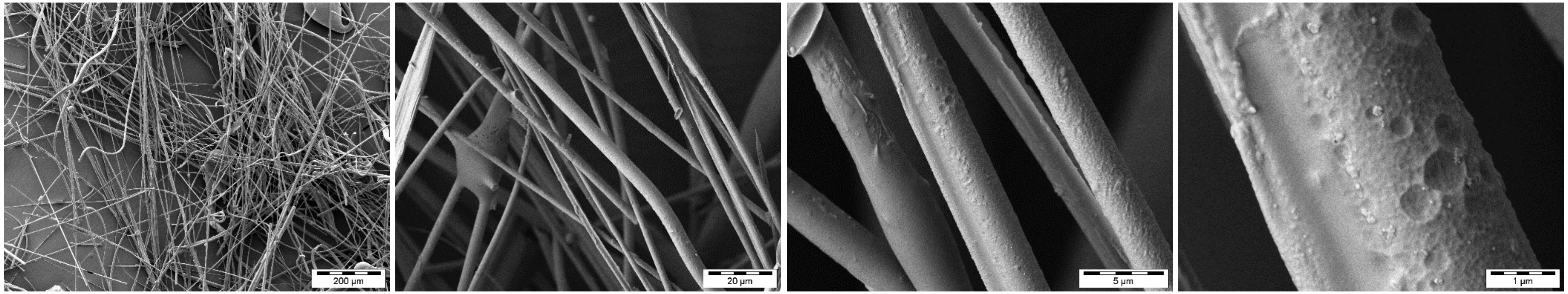

MMVF #24 (untreated reference)

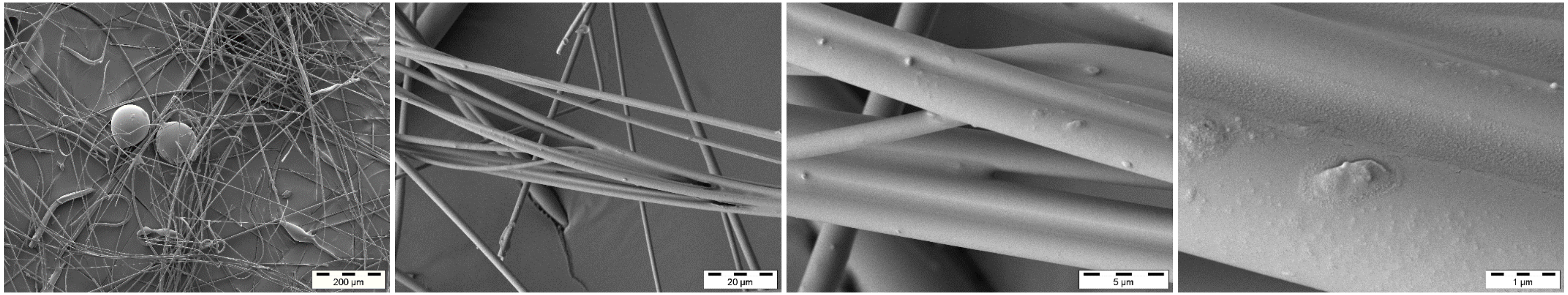

MMVF #24 (32 days pH 4.5)

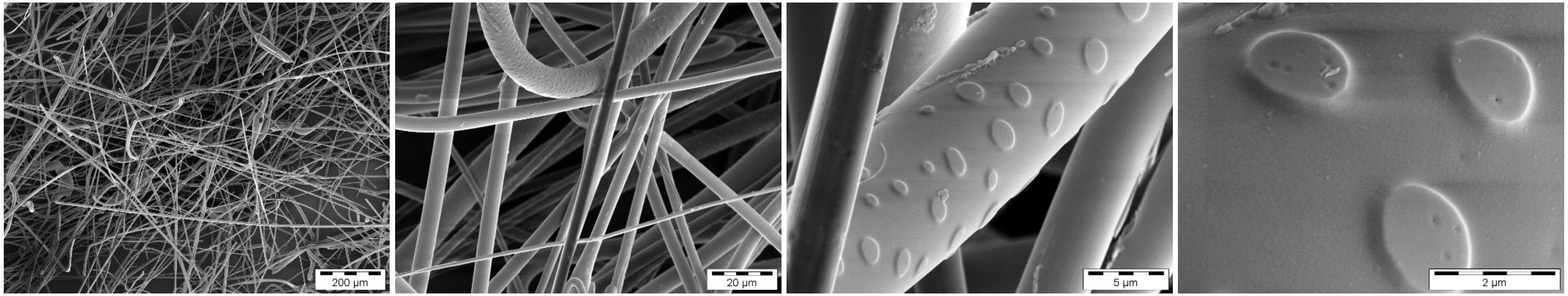

MMVF #26 (untreated reference)

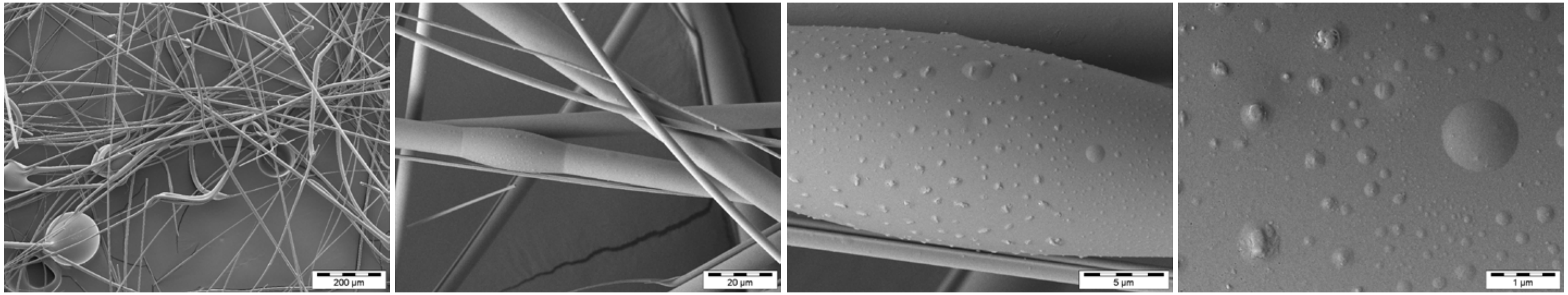

MMVF #26 (32 days pH 4.5)

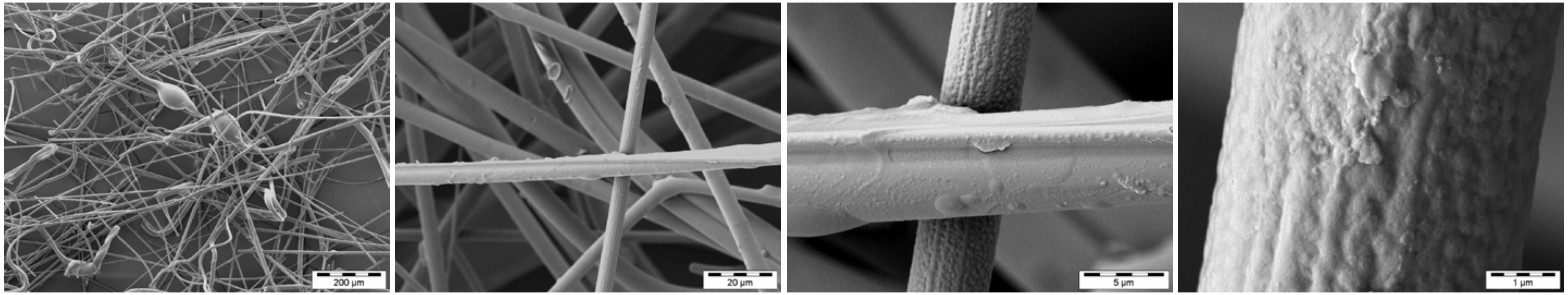

Supplement: Supplementary file 2 — Annex: extensive SEM before and after dissolution testing. (PDF 8398 kb) [file 12989_2017_210_MOESM2_ESM.pdf]
